# Supplementary material for: Spontaneous electric-polarization topology in confined ferroelectric nematics
Source: Nat Commun. 2022 Dec 17;13:7806. doi: 10.1038/s41467-022-35443-7 (PMC9759571; doi:10.1038/s41467-022-35443-7)
Supplement: Supplementary file 1 — Supplementary Information [file 41467_2022_35443_MOESM1_ESM.pdf]

Spontaneous electric-polarization topology in confined ferro-electric nematics

Jidan Yang[1]†, Yu Zou[1]†, Wentao Tang[1]†, Jinxing Li[1], Mingjun Huang[1,2]*, Satoshi Aya[1,2]*

#1. Supplementary Figures 1-15 ··2-16

#2. Materials and Methods ···17-20

#3. Supplementary Discussions 1-4 ··································21-23

#4. Supplementary Reference ···24

#1. Supplementary Figures 1-15

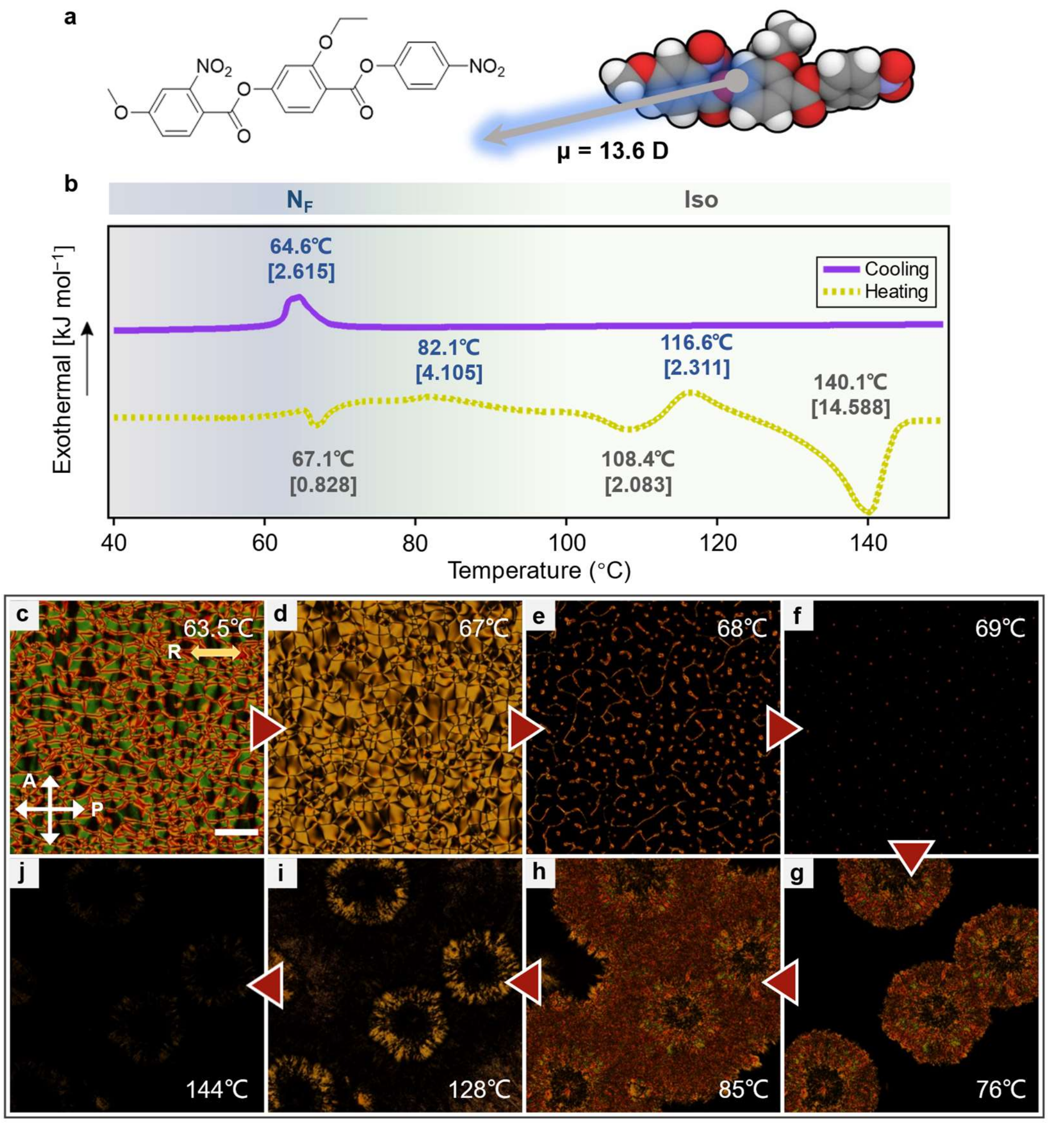

Supplementary Fig. 1. DSC curves and PLM images of the ferroelectric nematic. a, Chemical structure of RM-OC_2. **b**, DSC curves during cooling (violet solid line) and heating (gold dash line) at a scan rate of 3.0 K min^{-1}. Enthalpies of corresponding phase transitions are shown in the square brackets in the unit of kJ mol^{-1}. **c-j**, The PLM texture evolution of RM-OC_2 during heating. During the heating process, the system undergoes the crystallization after going into the Iso phase. Optical graphs are taken in an antiparallelly rubbed cell. Rubbing direction is indicated by the yellow arrow. Scale bar: 100 μm.

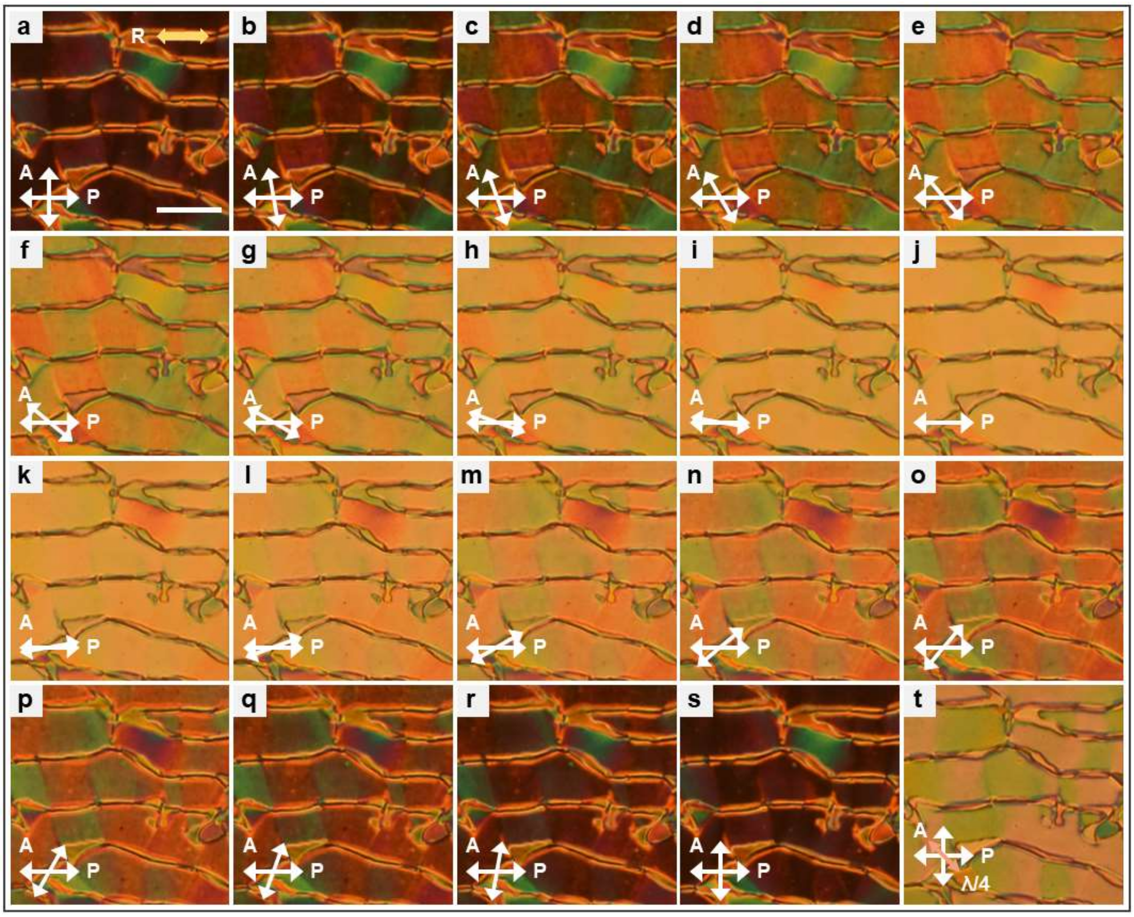

Supplementary Fig. 2. PLM observation of the N_F band-like texture of RM-OC_2. a, The LC cell was placed parallel to the P polarizer under the crossed polarizers. **b-s**, The analyzer is rotated by 10°-180° counterclockwise. **t**, PLM images of N_F band texture under crossed polarizers with a quarter-wave plate. The angle between the fast axis of the quarter-wave plate and the polarizer is 45°. The orange arrow indicates the fast axis of the quarter-wave plate. Optical graphs are taken in a parallel aligned cell. Rubbing direction is indicated by the yellow arrow. Scale bar: 50 μm. Temperature: 30°C.

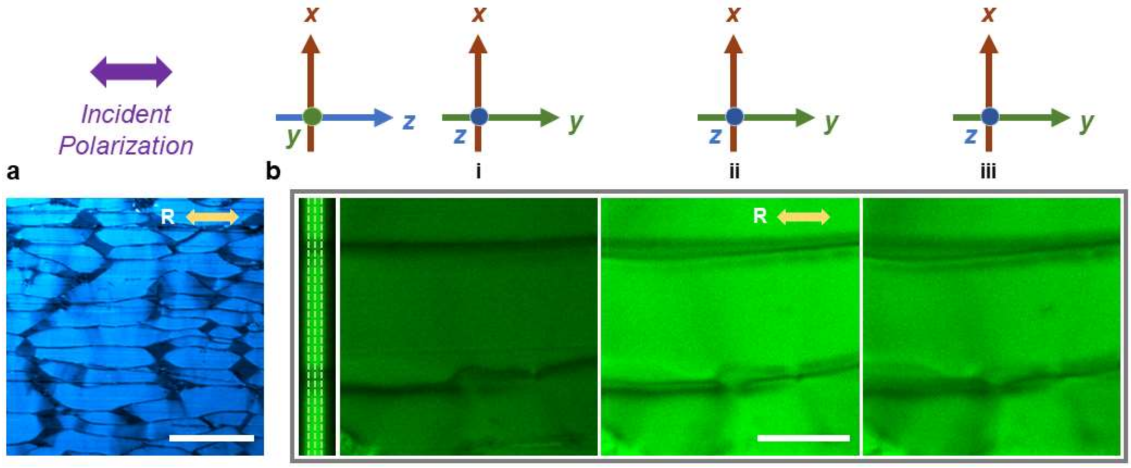

Supplementary Fig. 3. SHG confocal polarizing microscopy (SHG-CPM) image and FCPM images for N_F band-like texture in a parallel aligned cell. The purple arrow represents the linear polarization of the incident light. Rubbing direction is indicated by the yellow arrow. **a**, xy-cross-sectional SHG-CPM images of the N_F band-like texture of RM-OC_2. Scale bar, 50 µm. **b**, Cross-sectional FCPM images of the N_F band-like texture of RM-OC_2. xy-cross-sectional images are visualized by a linearly polarization at three different of XZ-cross-sectional positions: positions i, ii, iii correspond to bottom surface, middle plane and top surface, respectively. Scale bars, 5 µm. Temperature: 30 °C.

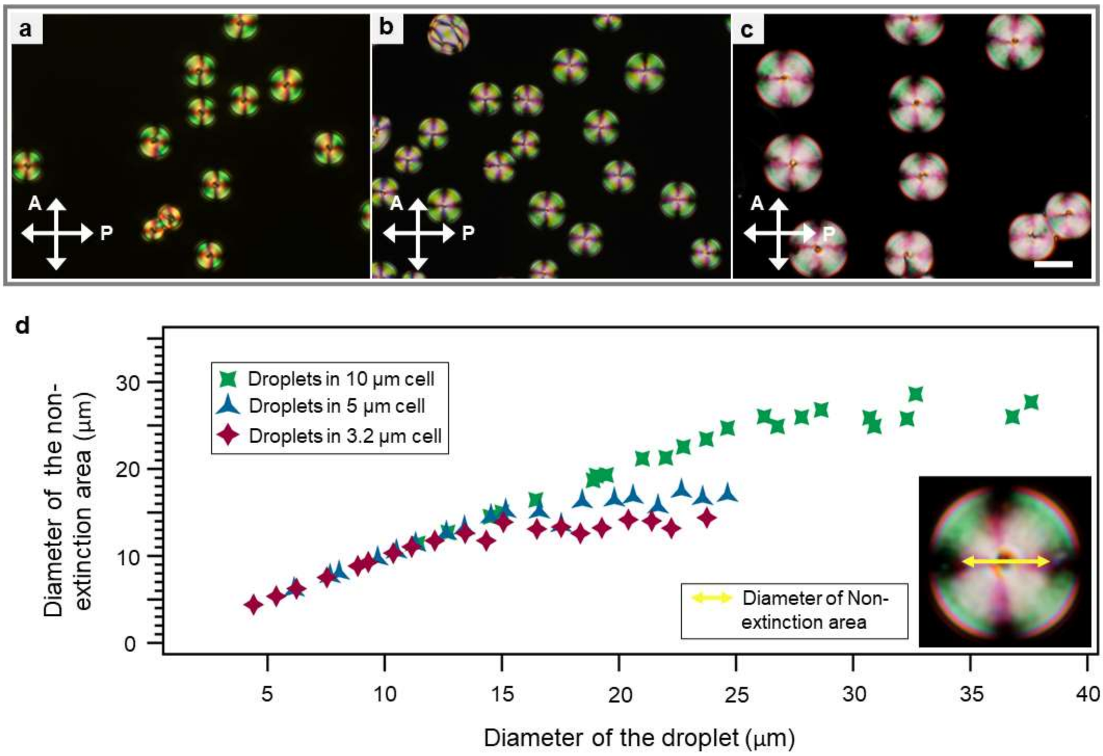

Supplementary Fig. 4. PLM characterization of N_F droplets. a-c, PLM images of the N_F droplets under crossed polarizers in homemade LC cells with the thickness of 3.2 μm (a), 5 μm (b), and 10 μm (c). Scale bar: 20 μm. **d**, The diagram showing the relationship between the non-extinction area in droplet center (indicated by a yellow arrow) and the diameter of the emerging N_F droplets in different film thicknesses.

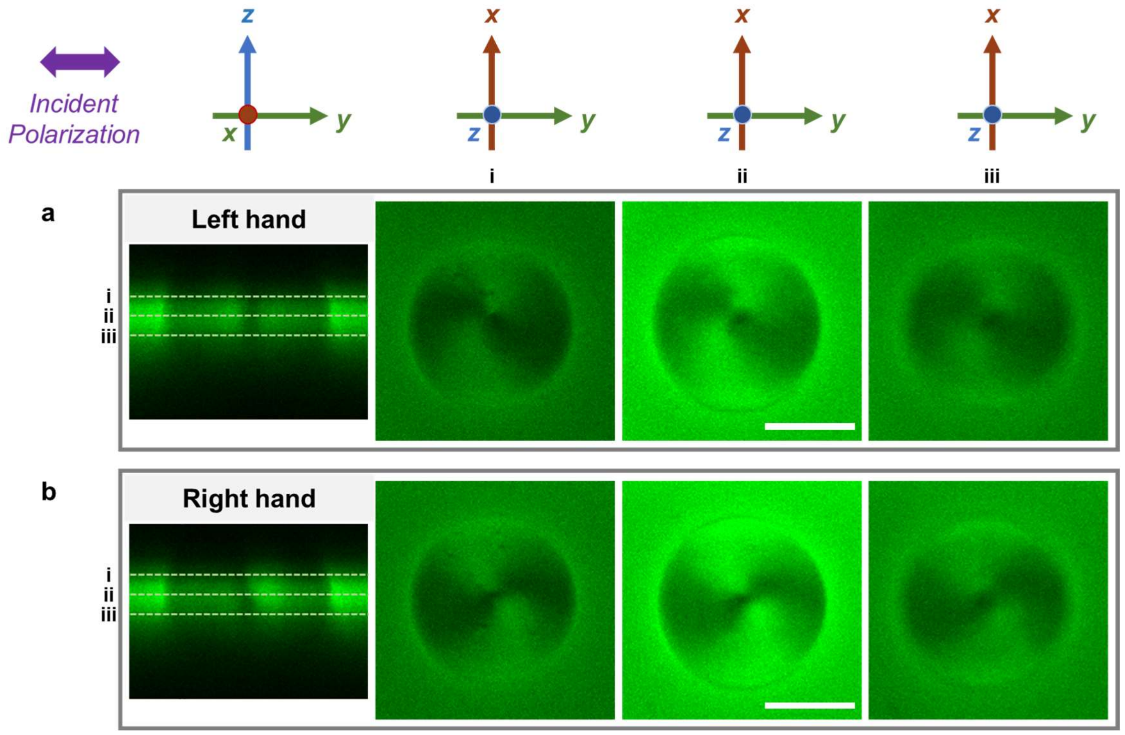

Supplementary Fig. 5. Fluorescence confocal polarizing microscopy (FCPM) images of N_F droplets with the opposite handedness. The purple arrow represents the linear polarization of the incident light. **a**,**b**, Cross-sectional FCPM images of the lefthand N_F droplet (a) and the right hand N_F droplet (b). XY- and YZ-cross-sectional images are visualized by a linearly polarization at three different positions. Scale bars, 10 µm.

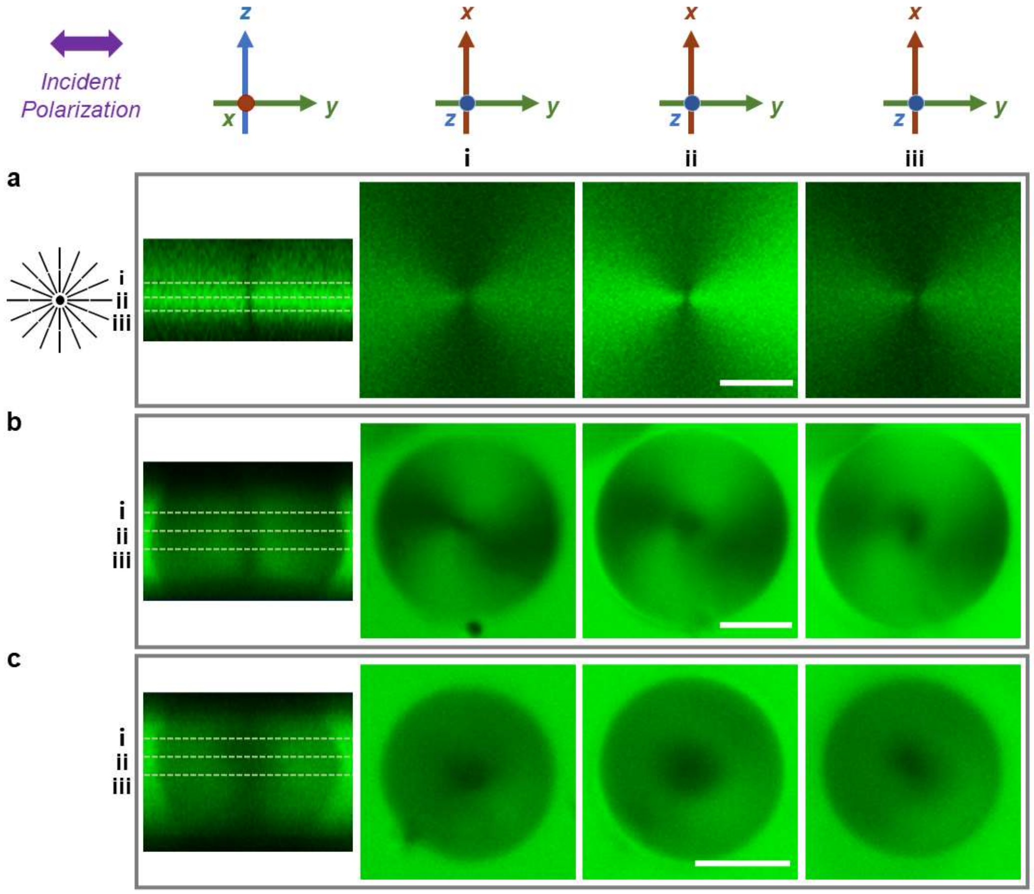

Supplementary Fig. 6. FCPM images for a photoaligned pattern and N_F droplets. The purple arrow represents the linear polarization of the incident light. **a**, XY- and YZ-cross-sectional FCPM images of a +1 disclination line that connects the top and bottom substrates in a 5 μm cell created by the photoalignment technique. We use a commercial 5CB doped with Nile Red (c = 0.05 %) to observe the line disclination under FCPM. The XY-cross-sectional images are visualized by the linearly polarization at three different positions, corresponding to i~iii. Scale bar, 40 μm. An objective lens with the numerical aperture NA=0.8 is used for the observations (Plan-ApoCHROMAT20x, Zeiss). **b**, XY-cross-sectional FCPM images of an N_F droplet of DIO (see Supplementary Discussion 2). The XY-cross-sectional images are visualized by a linearly polarization at three different positions, corresponding to i~iii in the YZ-cross-sections. Scale bar, 5 μm. An oil-immersion objective lens with the numerical aperture NA=1.4 is used for the observations (Plan-ApoCHROMAT63x, oil, Zeiss). A 5-μm cell with a planar alignment without rubbing is used for observation. **c**, XY-cross-sectional FCPM images of an N_F droplet of DIO. The XY-cross-sectional images are visualized by a circular polarization at three different positions, corresponding to i~iii. Scale bar, 5 μm.

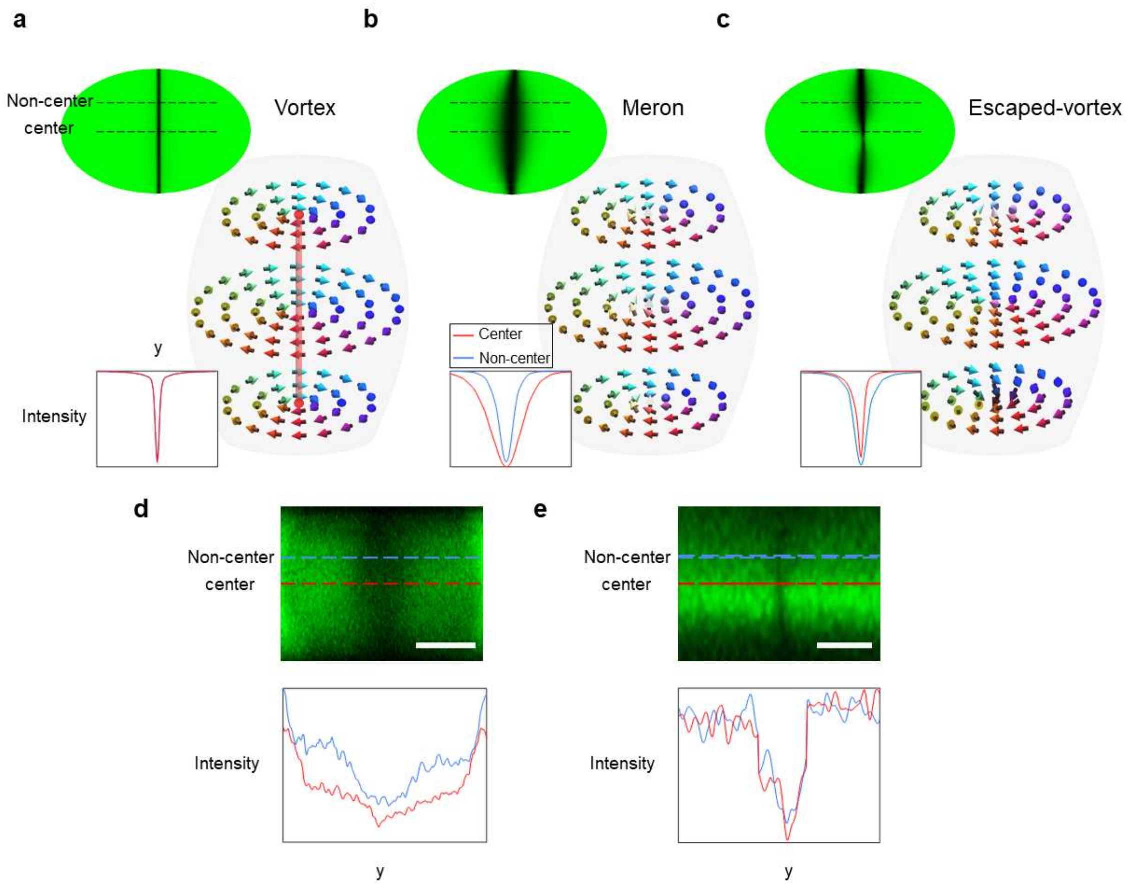

Supplementary Fig. 7. Fluorescence intensity profiles. a-c, Simulated structures of vortex (a), meron (b) and escaped vortex (c). Calculated YZ-cross-sections of FCPM images and signal profiles obtained from the simulated structures. The simulated FCPM images are visualized by a circular polarization. **d**,**e**, Experimental FCPM profiles of a N_F droplet (d) and the disclination line (e). The scale bar in (d) is 3 μm and in (e) is 20 μm. The experimental FCPM images (d) are visualized by a circular polarization and (e) are visualized by a linearly polarization. The red lines represent the intensity profile along the path in the center area of the droplet. The blue lines represent the intensity profile along the path in the non-center area of the droplet.

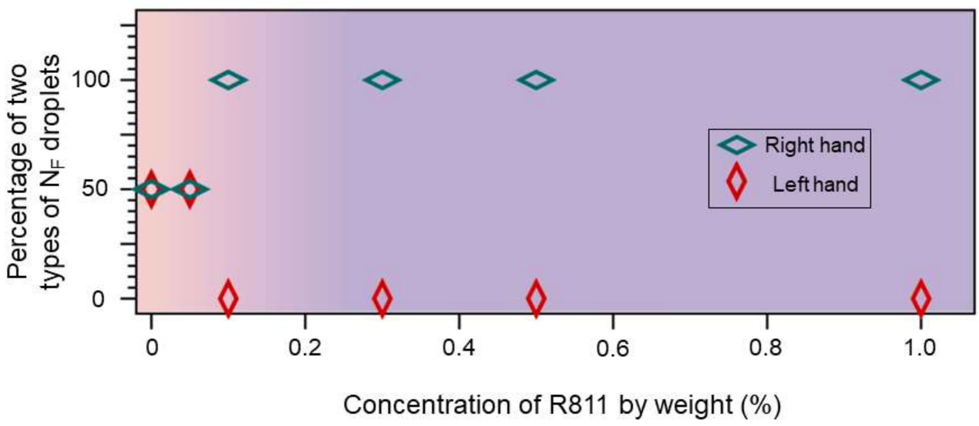

Supplementary Fig. 8. N_F droplet statistics. Percentage of two types of N_F droplets with the opposite handedness for RM-OC_2/R811 mixtures as a function of the weight percentage of R811.

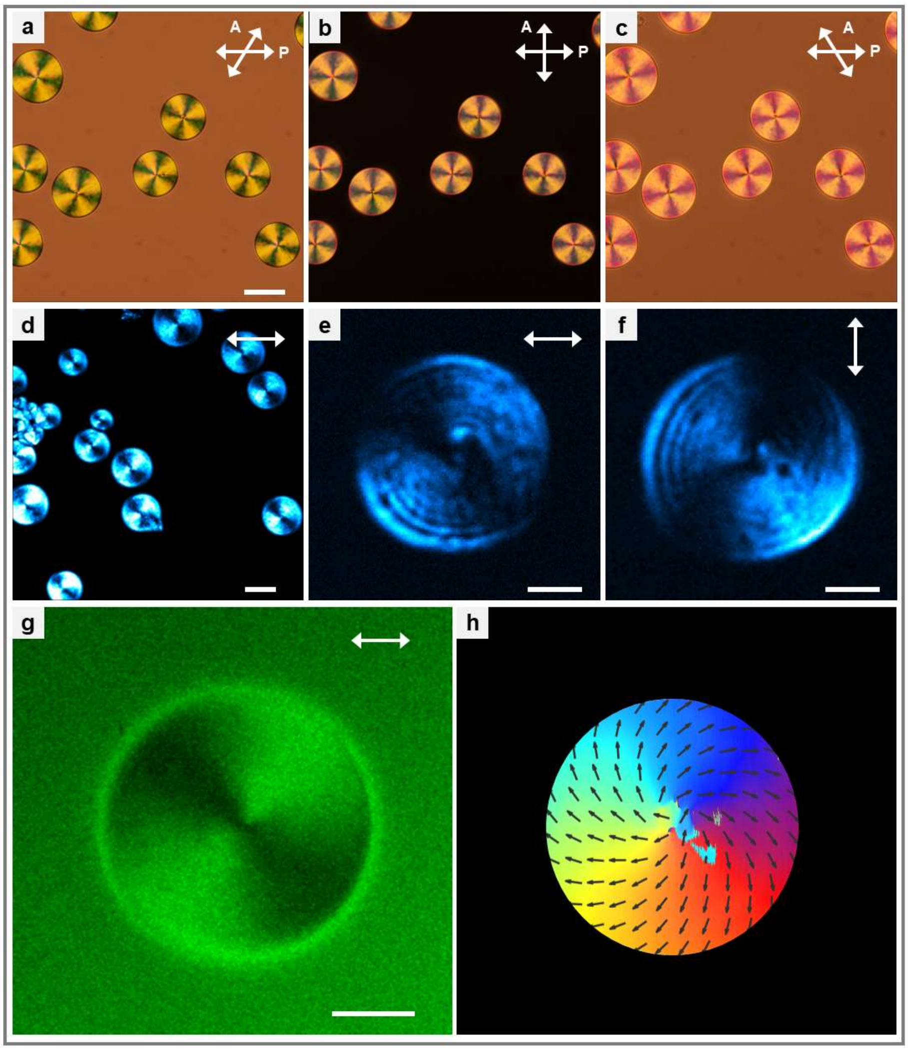

Supplementary Fig. 9. Chirality biasing of N_F droplets. a-c, PLM textures of an RM-OC_2 /S811 mixture (99.5/0.5 in wt%) taken under different combinations of polarizers. The analyzer is rotated 32° clockwise (a) and anticlockwise (c). Scale bar, 50 µm. **d**, XY-cross-sectional SHG-CPM image, Scale bar, 20 µm. **e**,**f**, SHG microscopy images with two different incident laser polarizations, Scale bars, 3 µm. **g**, XY-cross-sectional FCPM image visualized by a linearly polarization indicated by the white arrow, Scale bar, 10 µm. **h**, Reconstructed left-handed divergent polarization fields from SHG-I and FCPM data.

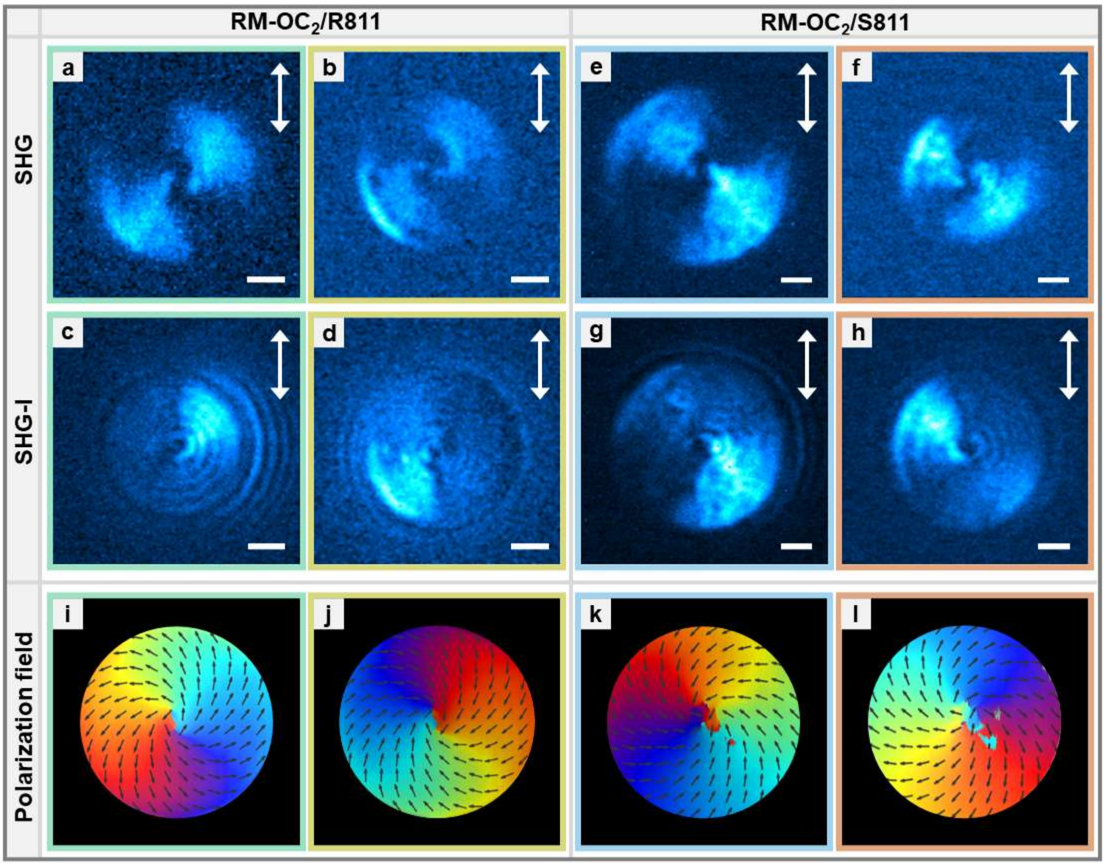

Supplementary Fig. 10. Polarization textures of chirality biased droplets probed by SHG and SHG-I microscopy. a-d, Experimental SHG microscopy (a, b) and SHG-I microscopy (c, d) observations of polarization textures of RM-OC_2/R811 mixture (99.5/0.5 in wt%). **e-h**, Experimental SHG microscopy (e, f) and SHG-I microscopy (g, h) observations of polarization textures of RM-OC_2/S811 mixture (99.5/0.5 in wt%). In SHG-I microscopy observations (c, d, g, h), the interference conditions are the same. Scale bars, 5 μm. **i-l**, Reconstructed polarization fields with right-handed divergent polarization field (i), left-handed convergent polarization field (g), right-handed convergent polarization field (k) and left-handed divergent polarization field (l).

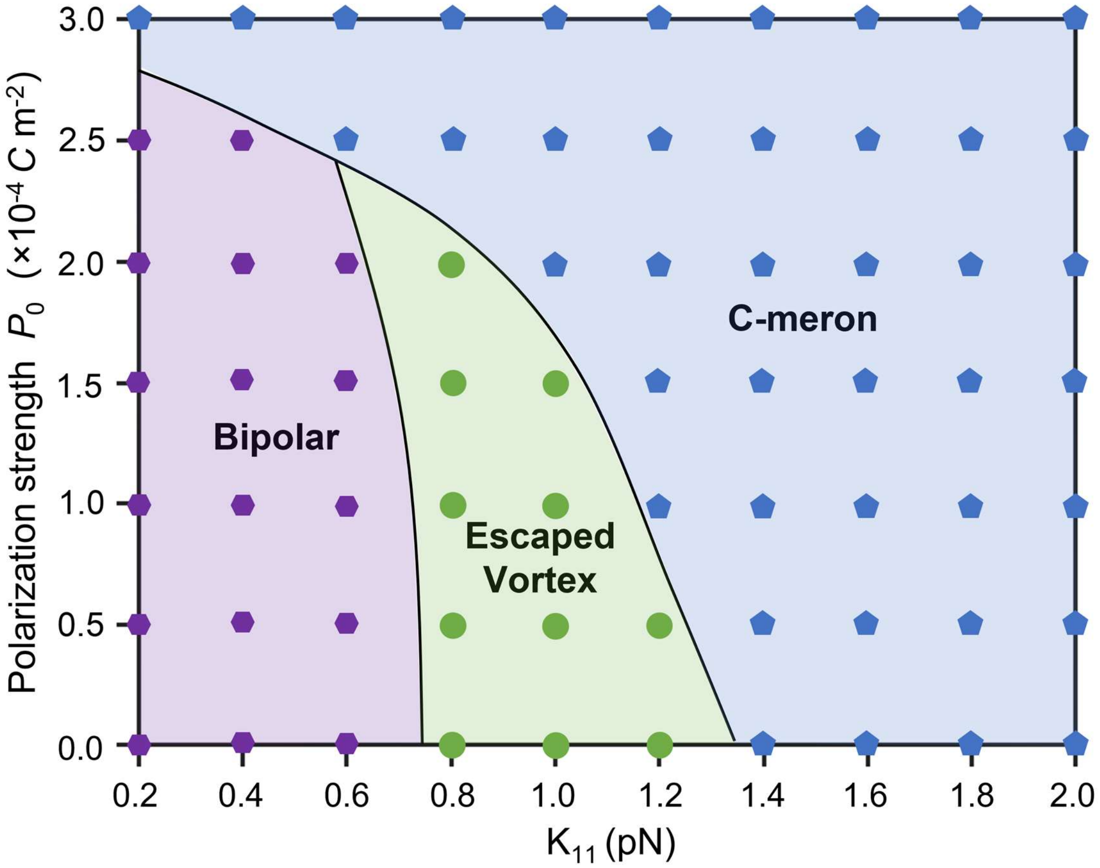

Supplementary Fig. 11. State diagram dependent on the splay modulus K_{11} and polarization strength P_0. We assume that the twist elastic modulus is equal to the bend elastic modulus, $K_{22} = K_{33} = 2$ pN. In the range of $P_0 < 2.5 \times 10^{-4}$C m^{-2}, as K_{11} decreases, the structure with the lowest energy changes from C-meron to escaped vortex structure, and then to bipolar structure. The reason for these transformations is that the above three structures contain more splay deformation in turn. However, in the region of larger polarity, $P_0 > 2.5 \times 10^{-4}$ C m^{-2}, the bipolar structure with two +1 point defects at its two poles will consume huge energy due to the sharp increase of dipolar interactions, so C-meron is again the most favorable even in the range of small K_{11}.

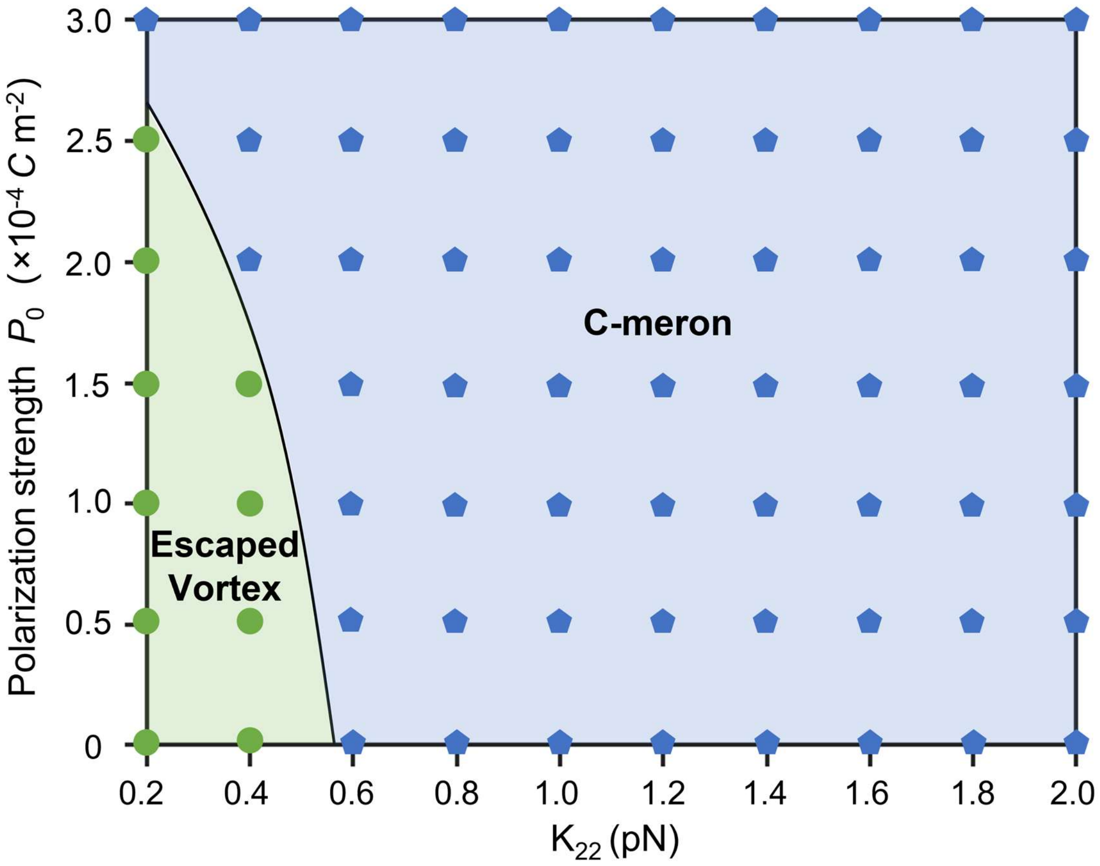

Supplementary Fig. 12. State diagram dependent on the twist modulus K_{22} and polarization strength $\boldsymbol{P_0}$. We assume that the splay elastic modulus is equal to the bend elastic modulus, $K_{11} = K_{33} = 2$ pN. In the range of $P_0 < 3.0 \times 10^{-4}$ C m^{-2}, as K_{22} decreases, the structure with the lowest energy changes from C-meron to escaped vortex structure, since the very small twist elastic modulus K_{22} breaks the symmetry of the system and thus spontaneously forms chiral structures in the non-chiral system. However, this effect is suppressed in the high polarity region, i.e. $P_0 = 3.0 \times 10^{-4}$ C m^{-2}, since the escaped vortex structure contains large energy from dipolar interaction near the -1 point defect in its center.

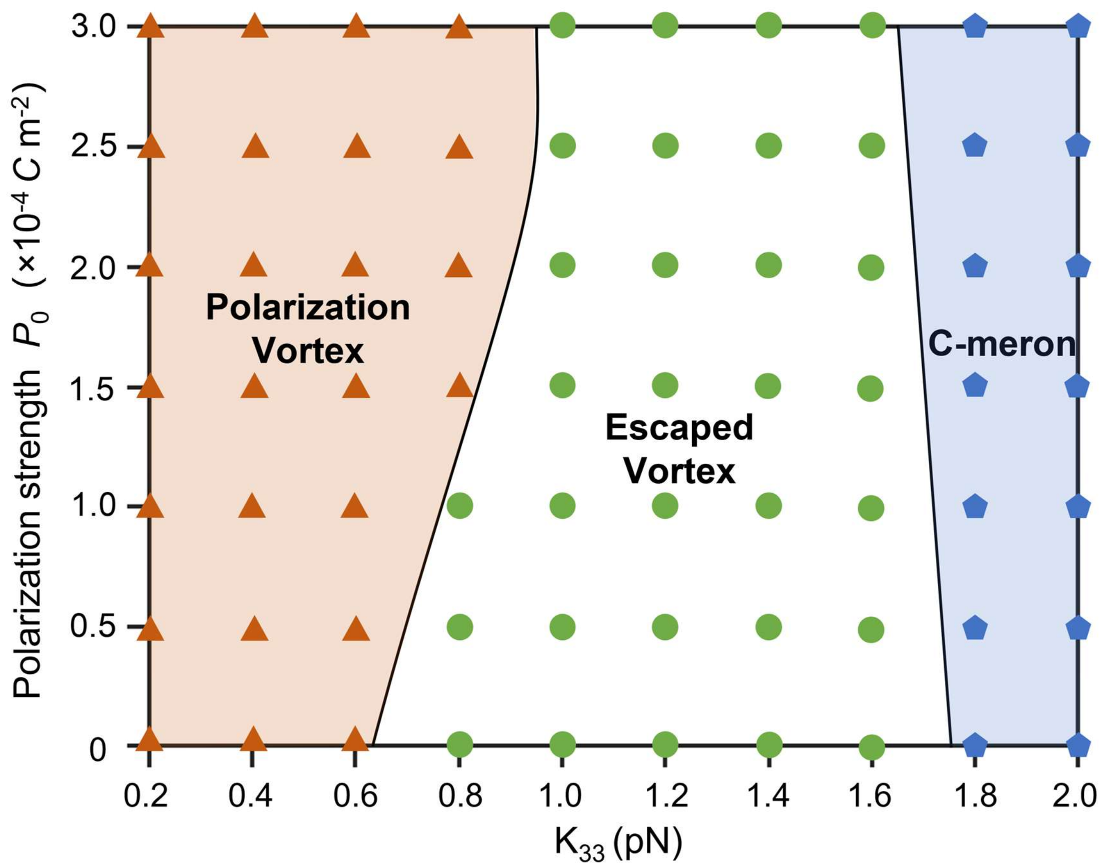

Supplementary Fig. 13. State diagram dependent on the bend modulus K_{33} and polarization strength P_0. We assume that the splay elastic modulus is equal to the twist elastic modulus, $K_{11} = K_{22} = 2$ pN. As the bend modulus K_{33} decreases, the structure with the lowest energy changes from C-meron to escaped vortex structure, and then to simple polarization vortex structure. The escaped vortex structure here is evolved from the simple polarization vortex structure which has a lot of bend deformation and has a defect line in its core. In order to reduce the total energy of structure, the LC molecules near the simple polarization vortex structure's core will escape along the z direction so that the line defect will degenerate into a point defect. The polarity affects the phase area of the three structures (high polarity shrinks the phase diagram area of the escaped vortex structure and increases that of the other two structures).

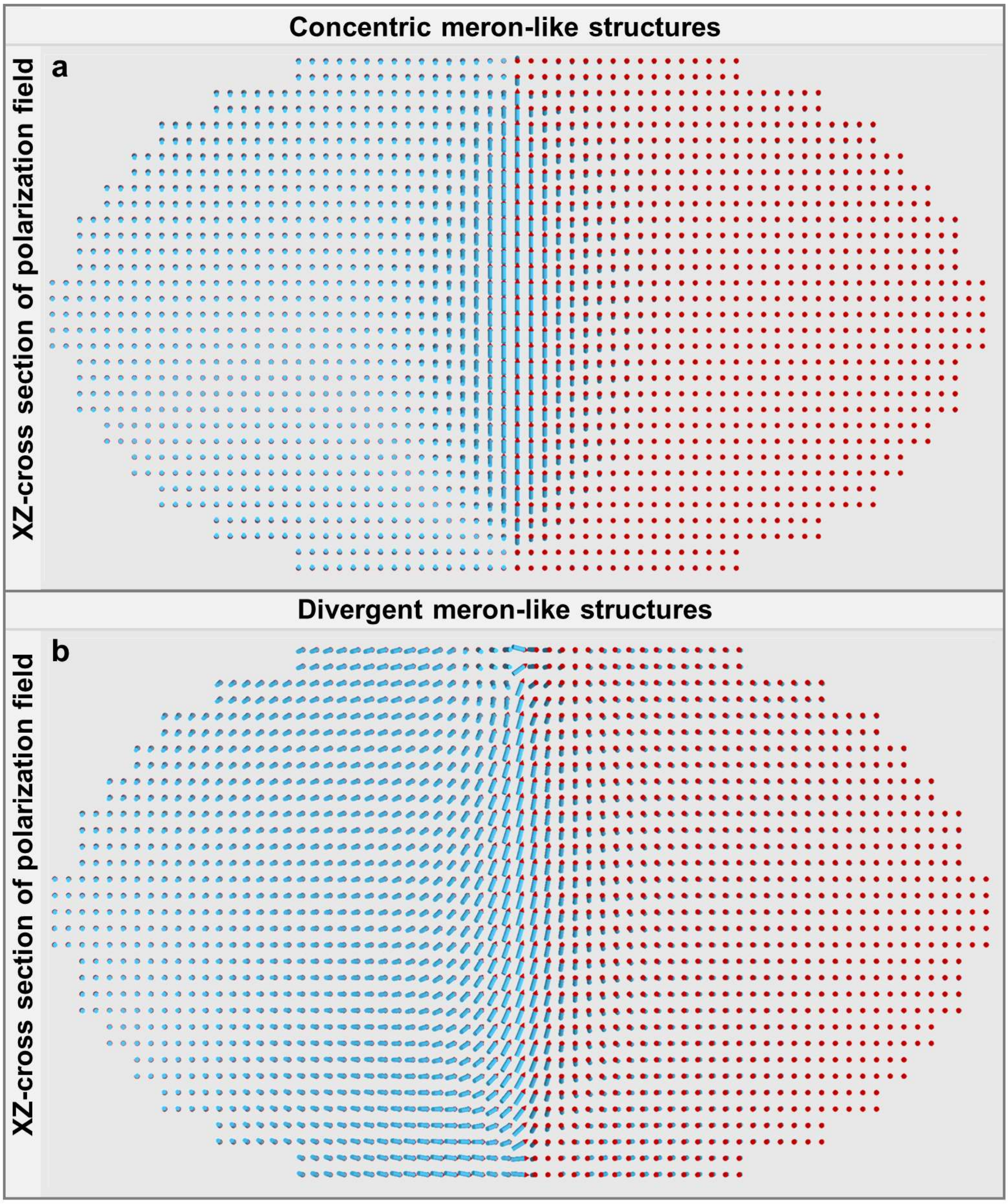

Supplementary Fig. 14. Comparison of simulation results between concentric meron-like structure and divergent meron-like structure in confined space. a,b, The XZ-cross-sectional polarization field of concentric meron-like structure (a) and divergent meron-like structure (b).

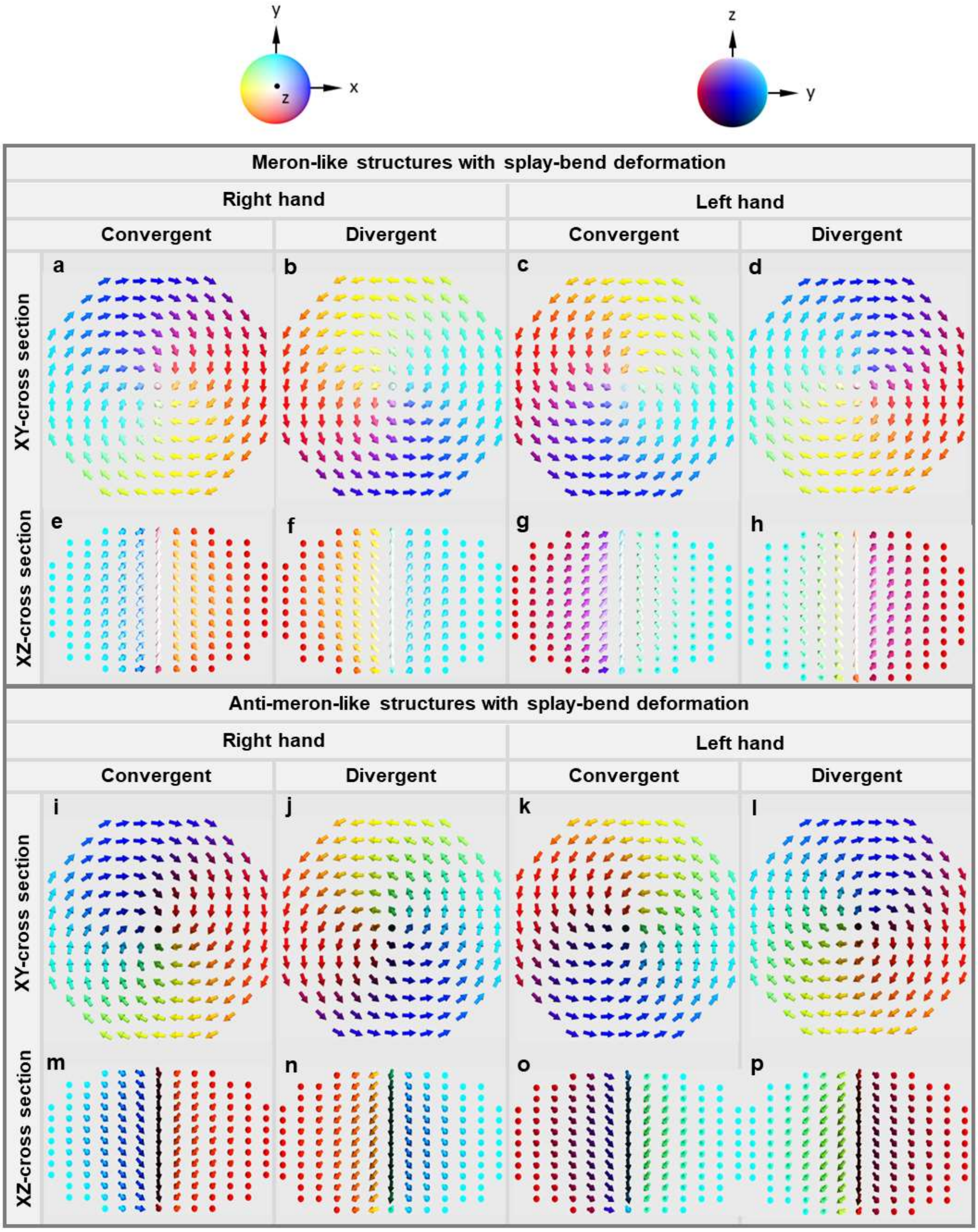

Supplementary Fig. 15. Numerical simulation results of possible divergent meron-like structure states and their corresponding anti-meron-like structures in confined space. a-h, Four types of polarization fields of divergent meron-like structure classified by the combination of handedness of the spirals and polarization orientation. **i-p**, Four types of polarization fields of the divergent (convergent) anti-meron-like structures classified by the combination of their handedness of the spirals handedness and polarization orientation.

#2. Materials and Methods

Materials

All commercial chemicals and solvents were used as received, unless stated otherwise. (R)-butan-2-ol, (S)-2-methylbutan-1-ol were obtained from Innochem. methyl 2-hydroxy-4-methoxybenzoate, 4-hydroxy-2-methoxybenzaldehyde, Sodium chlorite, Sodium dihydrogen phosphate were obtained from Energy Chemical. 4-nitrophenyl 4-hydroxybenzoate were obtained from Bidepharm. N-(3-dimethylaminopropyl)-N′-ethylcarbodiimide hydrochloride (EDC·HCl), and 4-dimethylaminopyridine (DMAP) were obtained from Sigma-Aldrich. Tetrahydrofuran (THF, Energy Chemical). Dichloromethane (DCM, Energy Chemical), Petroleum ether (PE, Energy Chemical) Ethyl acetate (EA, Energy Chemical), Methanol (MeOH, Energy Chemical, reagent grade), N,N-Dimethylformamide (DMF, Energy Chemical, reagent grade), Potassium carbonate, Potassium hydroxide were obtained from Sigma-Aldrich.

Characterizations of chemicals

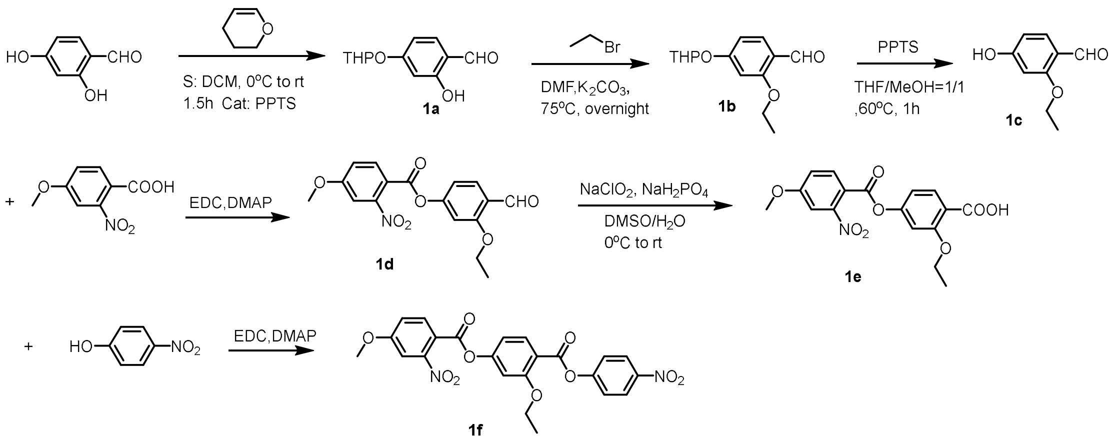

2-hydroxy-4-((tetrahydro-2H-pyran-2-yl)oxy)benzaldehyde (1a): A round bottom flask was charged with 2,4-dihydroxybenzaldehyde (2.6 g, 18.8 mmol), para-toluenesulfonic acid (0.196 g, 1.03 mol), and 100 mL diethyl ether forming a dispersion solution. The solution was degassed by bubbling nitrogen through for 5 min at ice bath. Then 3,4- Dihydro-2H-pyran (1.9 g, 2.06 mL, 22.6 mmol) was added dropwise via injector. The solution was slowly warmed to room temperature, and stirred under nitrogen for 5-6 h. The precipitate was then collected by filtration, washed with 50 mL diethyl ether (as little as possible), and dried in a vacuum oven. Yield: 2.3 g (55%); appearance: white powder. ^{1}H NMR (500 MHz, Chloroform-*d*) δ 11.36 (s, 1H), 9.70 (s, 1H), 7.42 (d, *J* = 8.6 Hz, 1H), 6.65 (dd, *J* = 8.6, 2.2 Hz, 1H), 6.61 (d, *J* = 2.2 Hz, 1H), 5.53 – 5.45 (m, 1H), 3.82 (td, *J* = 10.9, 10.5, 3.0 Hz, 1H), 3.66 – 3.60 (m, 1H), 1.86 (dt, *J* = 7.8, 3.8 Hz, 2H), 1.73 – 1.64 (m, 2H), 1.63 – 1.48 (m, 2H). ^{13}C NMR (126 MHz, Chloroform-*d*) δ 194.67, 164.44, 164.24, 135.38, 115.82, 109.49, 103.74, 96.31, 62.25, 30.00, 25.01, 18.48.

2-propoxy-4-((tetrahydro-2H-pyran-2-yl)oxy)benzaldehyde (1b): A 100 mL round bottom flask was charged with the compound **1** (2.22 g, 10 mmol), potassium carbonate (4.15 g, 30 mmol), and 30 mL DMF. The solution was degassed by bubbling nitrogen for 3 min. Then bromoethane (0.9 mL, 1.31 g, 12 mmol) was added via injector. The solution was heated at 75°C and vigorously stirred for overnight. The mixture was cooled to room temperature, 200 mL water poured, and extracted with EA. The organic phase was washed with water and brine, then dried with anhydrous $MgSO_4$. The solvent was removed by rotary evaporation, and dry-loaded onto a silica gel column for purification using EA/hexane as eluent. Yield: 2.41 g, 96.3%, appearance: colorless oil. ^{1}H NMR (400

MHz, Chloroform-*d*) δ 10.34 (s, 1H), 7.78 (d, *J* = 8.7 Hz, 1H), 6.75 – 6.53 (m, 2H), 5.51 (t, *J* = 3.1 Hz, 1H), 4.12 (qd, *J* = 7.0, 2.3 Hz, 2H), 3.93 – 3.79 (m, 1H), 3.70 – 3.56 (m, 1H), 2.06 – 1.93 (m, 1H), 1.87 (dt, *J* = 7.6, 3.6 Hz, 2H), 1.74 – 1.64 (m, 2H), 1.61 (dt, *J* = 12.1, 3.9 Hz, 1H), 1.47 (t, *J* = 7.0 Hz, 3H). ^{13}C NMR (101 MHz, Chloroform-*d*) δ 188.60, 163.70, 163.11, 130.02, 119.42, 108.44, 100.35, 96.14, 64.15, 62.04, 30.06, 25.03, 18.40, 14.58.

2-ethoxy-4-hydroxybenzaldehyde (1c): A round bottom flask was charged with **1b** (2.0 g, 7.99 mmol), PPTS (2.01 g, 7.99mmol), 20 mL THF, and 20 mL ethanol. The solution was heated at 60 °C and monitored by TLC (6-48 h). The solvent was removed under reduced pressure. Water (40 mL) was added to the residue, and the product was extracted with DCM (3 × 50 mL). The organic phase was dried by anhydrous $MgSO_4$. The solvent was removed and the residue was purified by chromatography. The product was then concentrated and dried in the vacuum oven. Yield: 1.28 g (96.4%); appearance: white powder. ^{1}H NMR (400 MHz, Chloroform-*d*) δ 10.32 (s, 1H), 7.77 (d, *J* = 8.4 Hz, 1H), 6.49 – 6.38 (m, 2H), 5.84 (s, 1H), 4.11 (q, *J* = 6.9 Hz, 2H), 1.47 (t, *J* = 7.0 Hz, 3H). ^{13}C NMR (101 MHz, Chloroform-*d*) δ 188.51, 163.58, 162.70, 130.62, 119.01, 99.41, 64.21, 14.57.

3-ethoxy-4-formylphenyl 4-methoxy-2-nitrobenzoate (1d): A round bottom flask was charged with **1c** (1.2 g, 7.22 mmol), 4-methoxy-2-nitrobenzoic acid (1.57 g, 7.94 mmol), EDC (1.68 g, 10.83 mmol), N, N-dimethylaminopyridine (0.089 g, 0.722 mmol), then 50 mL dichloromethane solvent was added and cooled to 0 °C and flashed with nitrogen. The solution was stirred at 0 °C for 1 h then at room temperature for 17 hr. The solution was stripped of solvent by rotary evaporation, and dry-loaded onto a silica gel column for purification using dichloromethane/hexane as eluent. Yield: 1.95g (78.2%); appearance: white powder. ^{1}H NMR (400 MHz, Chloroform-*d*) δ 10.45 (s, 1H), 7.91 (t, *J* = 8.6 Hz, 2H), 7.38 (d, *J* = 2.4 Hz, 1H), 7.21 (dd, *J* = 8.7, 2.5 Hz, 1H), 6.88 (p, *J* = 3.1, 2.6 Hz, 2H), 4.17 (q, *J* = 7.0 Hz, 2H), 3.96 (d, *J* = 1.6 Hz, 3H), 1.50 (t, *J* = 7.0 Hz, 3H). ^{13}C NMR (101 MHz, Chloroform-*d*) δ 188.83, 162.94, 162.60, 162.39, 156.27, 132.48, 130.58, 129.73, 123.01, 117.88, 117.28, 113.70, 109.74, 106.02, 64.66, 56.34, 14.51.

2-ethoxy-4-((4-methoxy-2-nitrobenzoyl)oxy)benzoic acid (1e): Sodium dihydrogen phosphate (2.64 g, 22.01 mmol) and sodium chlorite (1.74 g, 19.26 mmol) were dissolved in water (30 mL) and slowly added to a stirred solution of compound **1d** (1.9

g, 5.5 mmol) in DMSO (40 mL) by constant pressure dropping funnel at 0 °C. The mixture was allowed to warm to room temperature and stir for 6 hours. The mixture was diluted with water and solid $NaHCO_3$ was added to adjust the pH of the solution to 9. The solution was washed with ethyl acetate, then the pH was adjusted to 4 by the addition of 1M HCl solution and extracted with ethyl acetate. The combined organic extracts ware washed with brine, dried ($NaSO_4$). The solvent was removed by rotary evaporation, and dry-loaded onto a silica gel column for purification using EA/hexane as eluent. Yield: 1.92 g, 96.6%, appearance: white powder. ^{1}H NMR (500 MHz, DMSO-d_6) δ 12.60 (s, 1H), 8.09 (d, *J* = 8.7 Hz, 1H), 7.70 (d, *J* = 8.4 Hz, 1H), 7.64 (d, *J* = 2.5 Hz, 1H), 7.38 (dd, *J* = 8.7, 2.5 Hz, 1H), 7.01 (d, *J* = 2.1 Hz, 1H), 6.83 (dd, *J* = 8.4, 2.1 Hz, 1H), 4.05 (q, *J* = 6.9 Hz, 2H), 3.91 (s, 3H), 1.29 (t, *J* = 6.9 Hz, 3H). ^{13}C NMR (126 MHz, DMSO-d_6) δ 167.19, 163.68, 162.37, 159.06, 153.91, 151.53, 133.45, 132.39, 120.25, 118.49, 115.51, 113.57, 110.39, 107.77, 65.01, 57.23, 14.96.

3-ethoxy-4-((4-nitrophenoxy)carbonyl)phenyl 4-methoxy-2-nitrobenzoate (1f): A round bottom flask was charged with **1e** (1.8 g, 4.98 mmol), 4-nitrophenol (0.76 g, 5.48 mmol), EDC (1.16 g, 7.47 mmol), N, N-dimethylaminopyridine (36.5 mg, 0.3 mmol), then 50 mL dichloromethane solvent was added and cooled to 0 °C and flashed with nitrogen. The solution was stirred at 0 °C for 1 h then at room temperature for 17 hr. The solution was stripped of solvent by rotary evaporation, and dry-loaded onto a silica gel column for purification using dichloromethane/hexane as eluent. Yield: 1.86g (77.4%); appearance: white powder. ^{1}H NMR (500 MHz, Chloroform-*d*) δ 8.35 – 8.24 (m, 2H), 8.12 – 8.04 (m, 1H), 7.92 (d, *J* = 8.6 Hz, 1H), 7.45 – 7.34 (m, 3H), 7.20 (dd, *J* = 8.6, 2.5 Hz, 1H), 6.97 – 6.87 (m, 2H), 4.17 (q, *J* = 7.0 Hz, 2H), 3.95 (s, 3H), 1.48 (t, *J* = 7.0 Hz, 3H). ^{13}C NMR (126 MHz, Chloroform-*d*) δ 163.07, 162.69 (d, *J* = 3.7 Hz), 161.04, 162.70, 162.67, 155.94, 155.64, 150.87, 145.38, 133.78, 132.57, 125.30, 122.77, 117.97, 117.31, 116.02, 113.28, 109.84, 106.85, 65.15, 56.42, 14.63.

#3. Supplementary Discussions 1-4

Supplementary Discussion 1. The phase behaviors of RM-OC_2 are shown in Figures 2b-d and Supplementary Figures 1c-j. The phase behavior upon the heating process is not simply mirror of that of the cooling process, exhibiting the phase sequence of N_F-[67.1 ˚C]-Iso-[82.1 ˚C]-unknown crystal X1-[105-120 ˚C]- unknown crystal X2-[140.1 ˚C]-Iso. During the temperature increases from 63 °C to 150 °C, the band-like texture of N_F phase gradually melts into the Iso phase (Supplementary Figs. 1c-f), and some crystal nuclei emerge. The crystalline phase X1 is replaced by another crystalline phase X2 at high temperatures, and then disappears upon further heating at 140.1 ˚C (Supplementary Figs. 1g-j). The transition process suggests the metastability of the system. At low temperatures on cooling, though crystal is the most stable state, the slow nucleation and growth kinetics does not allow the system crystalized at the observation time scale (up to days). However, we can observe very small non-growing nucleus of crystal phase. Upon heating, the thermal perturbation provides fluctuation to the system to transit to the most-stable crystal phase. As shown in Supplementary Figs. 1f-g, the domains readily crystallized upon heating can be attributed to the seeding effect from the remaining nucleus. This is also clearly observed in the DSC measurement (Supplementary Fig. 1).

Supplementary Discussion 2. Figure 2a demonstrates the phase behaviors of RM-OC_2. The temperature of the Iso-N_F transition is 64.6 °C and it exceeds the temperature limit of the oil immersion objective. Therefore, to obtain high resolution FCPM images, we choose an alternative material that shows the same N_F droplets but at low temperatures. We synthesized two pure and stable conformers of DIO: *trans*- and *cis*- conformers, and mixed the *cis* conformer into the *trans* conformer. The synthesis of pure stereoisomers of DIO is described in Ref. 1. Under the mixing ratio of *trans*/*cis* conformers = 6/4, a direct Iso-N_F phase transition occurs at 42 °C. The FCPM images of the N_F droplets are captured

at the temperature (Supplementary Figs. 6b,c). The result is fully consistent with that of RM-OC$_2$ (Supplementary Fig. 5). This confirms the general incidence of the electric polarization meron-like structures.

Supplementary Discussion 3. As seen from the FCPM under a circular polarized pumping light, a decrease of fluorescence occurs near the droplet center, while the other areas show a nearly constant intensity of fluorescence. In the YZ-cross-sectional profile (Supplementary Fig. 6c) the fluorescence intensity in the droplet center appears as a broaden black cylinder along the surface normal. This suggests the director field exhibits either a significant out-of-plane tilting towards the center (i.e. homeotropic-like in the center) or a disclination line. To determine the plausible structures, we compare the FCPM images of a disclination line with those of N$_F$ droplets. The line defect is constructed by employing a photo-alignment cell. The substrates are manufactured into patterns of +1 hedgehog-type defects. 5CB doped with Nile Red (c = 0.05 wt%) is filled into the cell. A +1 line disclination forms, connecting to both surfaces of the substrates. The dark region of the FCPM images of the line disclination (Supplementary Fig. 6a) is apparently extremely sharp and thin even when one deliberately uses a low-NA objective. It is not consistent with the broaden black cylinder in N$_F$ droplets (Supplementary Fig. 6b), suggesting that the disclination line is absent and the defects in the N$_F$ droplets. Therefore, at this stage, the concentric director fields might be either meron-like structure or escaped vortex structure.

To further differentiate the topology, we simulate the cross-sectional fluorescence profiles of these three structures and calculate the fluorescence intensity along paths in the center area and in the non-center areas (Supplementary Figs. 7a-c). The same profiling treatments are employed to the experimental FCPM images to obtain the signal profile (Supplementary Fig. 7d). From the signal profile of the escaped vortex (Supplementary

Fig. 7c), it is seen that, the signal profile along the center path is the thinnest, and outsides exhibit symmetrically identical signal distribution with respect to the center line. It is because the polarizations in the center line of the vortex lie in-plane without severe tilting. Predicted from the simulated meron-like structure (Supplementary Figs. 14-15), as the singularity is missing in the droplet center, the decrease of the intensity in the droplet center is due to the out-of-plane tilting of the polarizations. Since the N_F-glass interface poses a degenerate anchoring, if the directors are closer to the substrates, the directors therefore exhibit less out-of-plane tilting so the fluorescence intensity decreases will be less in the area closer to the substrates than in the midplane. This feature is represented in the fluorescence cross section profile and signal profile of the meron-like structure: the extinction area in the droplet center is larger than that in the non-center area (Supplementary Fig. 7b). Comparing the numerical FCPM images with the experimental results, it is clear that the N_F droplets demonstrate the meron-like structure topology (Supplementary Fig. 7d).

Supplementary Discussion 4. According to our observation, RM-OC_2 directly transits from isotropic to N_F with an intermittent co-existence (Figs. 2b,c). When the sample is further cooled down, the droplets merge and transit to a band-like texture, where the line disclinations run mainly along the rubbing direction (Fig. 2d). The corresponding director and polarization field can be directly visualized by SHG microscopy. Supplementary Fig. 3a demonstrates a large-area 2D SHG confocal polarizing microscopy (SHG-CPM) image of the band texture for the cell midplane during the cooling at 30 °C. In each domain, the SH signal shows the maximum intensity when the polarization is parallel to rubbing direction. This means the polarization points along the rubbing direction. Similar to Ref. (1) [Li, J., Nishikawa, H., Kougo, J., *et al. Sci. Adv.* 2021, **7**(17): eabf5047.], the SHG interferometry reveals that the neighboring domains exhibit opposite polarity.

#4. Supplementary Reference

1. Li J., *et al.* Development of ferroelectric nematic fluids with giant ε dielectricity and nonlinear optical properties. *Sci Adv* **7**, eabf5047 (2021).
