# Supplementary material for: Spontaneous electric-polarization topology in confined ferroelectric nematics
Source: Nat Commun. 2022 Dec 17;13:7806. doi: 10.1038/s41467-022-35443-7 (PMC9759571; doi:10.1038/s41467-022-35443-7)
Supplement: Supplementary file 3 — Reporting Summary [file 41467_2022_35443_MOESM3_ESM.pdf]

nature research

Corresponding author(s): Mingjun Huang; Satoshi Aya

Lasing Reporting Summary

Nature Research wishes to improve the reproducibility of the work that we publish. This form is intended for publication with all accepted papers reporting claims of lasing and provides structure for consistency and transparency in reporting. Some list items might not apply to an individual manuscript, but all fields must be completed for clarity.

For further information on Nature Research policies, including our data availability policy, see Authors & Referees.

ü Experimental design

Please check: are the following details reported in the manuscript?

1. Threshold

Plots of device output power versus pump power over a wide range of values indicating a clear threshold

☐ Yes — *State where this information can be found in the text.*
☒ No — In our experiment, the most important point is the wavelength of the laser to produce second harmonic geneation. It is not necessary for testing the threshold of laser.

2. Linewidth narrowing

Plots of spectral power density for the emission at pump powers below, around, and above the lasing threshold, indicating a clear linewidth narrowing at threshold

☐ Yes — *State where this information can be found in the text.*
☒ No — Not necessary for fabrication and testing the laser.

Resolution of the spectrometer used to make spectral measurements

☐ Yes — *State where this information can be found in the text.*
☒ No — *Explain why this information is not reported/not relevant.*

3. Coherent emission

Measurements of the coherence and/or polarization of the emission

☒ Yes — Figures 3h and 3j, Figures 4d to 4j, Figures 5d to 5g.
☐ No — *Explain why this information is not reported/not relevant.*

4. Beam spatial profile

Image and/or measurement of the spatial shape and profile of the emission, showing a well-defined beam above threshold

☒ Yes — See Methods of SHG and SHG interference (SHG-I) measurement.
☐ No — *Explain why this information is not reported/not relevant.*

5. Operating conditions

Description of the laser and pumping conditions
Continuous-wave, pulsed, temperature of operation

☒ Yes — See Methods of SHG and SHG interference (SHG-I) measurement.
☐ No — *Explain why this information is not reported/not relevant.*

Threshold values provided as density values (e.g. W cm^{-2} or J cm^{-2}) taking into account the area of the device

☐ Yes — *State where this information can be found in the text.*
☒ No — Not necessary for testing the threshold values of laser.

6. Alternative explanations

Reasoning as to why alternative explanations have been ruled out as responsible for the emission characteristics
e.g. amplified spontaneous, directional scattering; modification of fluorescence spectrum by the cavity

☒ Yes — Yes they have been ruled out.
☐ No — *Explain why this information is not reported/not relevant.*

7. Theoretical analysis

Theoretical analysis that ensures that the experimental values measured are realistic and reasonable
e.g. laser threshold, linewidth, cavity gain-loss, efficiency

☐ Yes — See Methods and Pages 6 and 7.
☒ No — *Explain why this information is not reported/not relevant.*

8. Statistics

Number of devices fabricated and tested

☒ Yes — More than 5 different liquid crystal cell with same sample were tested.
☐ No — *Explain why this information is not reported/not relevant.*

Statistical analysis of the device performance and lifetime (time to failure)

☐ Yes — *State where this information can be found in the text.*
☒ No — There was no statistical analysis on this device.
