# Supplementary material for: Spontaneous electric-polarization topology in confined ferroelectric nematics
Source: Nat Commun. 2022 Dec 17;13:7806. doi: 10.1038/s41467-022-35443-7 (PMC9759571; doi:10.1038/s41467-022-35443-7)
Supplement: Supplementary file 4 — Source Data [file 41467_2022_35443_MOESM4_ESM.zip › Source Data/Source Data Fig.6g/Read me.docx]

The data in this folder are the numerical simulation results for each point in Fig. 6g.

The names of subfolders in the main folder read (a, b), which means each simulation condition with a combination of the polarization strength and the elasticity ratio, i.e. P_0_=a×10^-4^ C m^-2^ and *K*_11_/*K*_22_=b".
