# Supplementary figures and images for: Spontaneous electric-polarization topology in confined ferroelectric nematics
Source: Nat Commun. 2022 Dec 17;13:7806. doi: 10.1038/s41467-022-35443-7 (PMC9759571; doi:10.1038/s41467-022-35443-7)

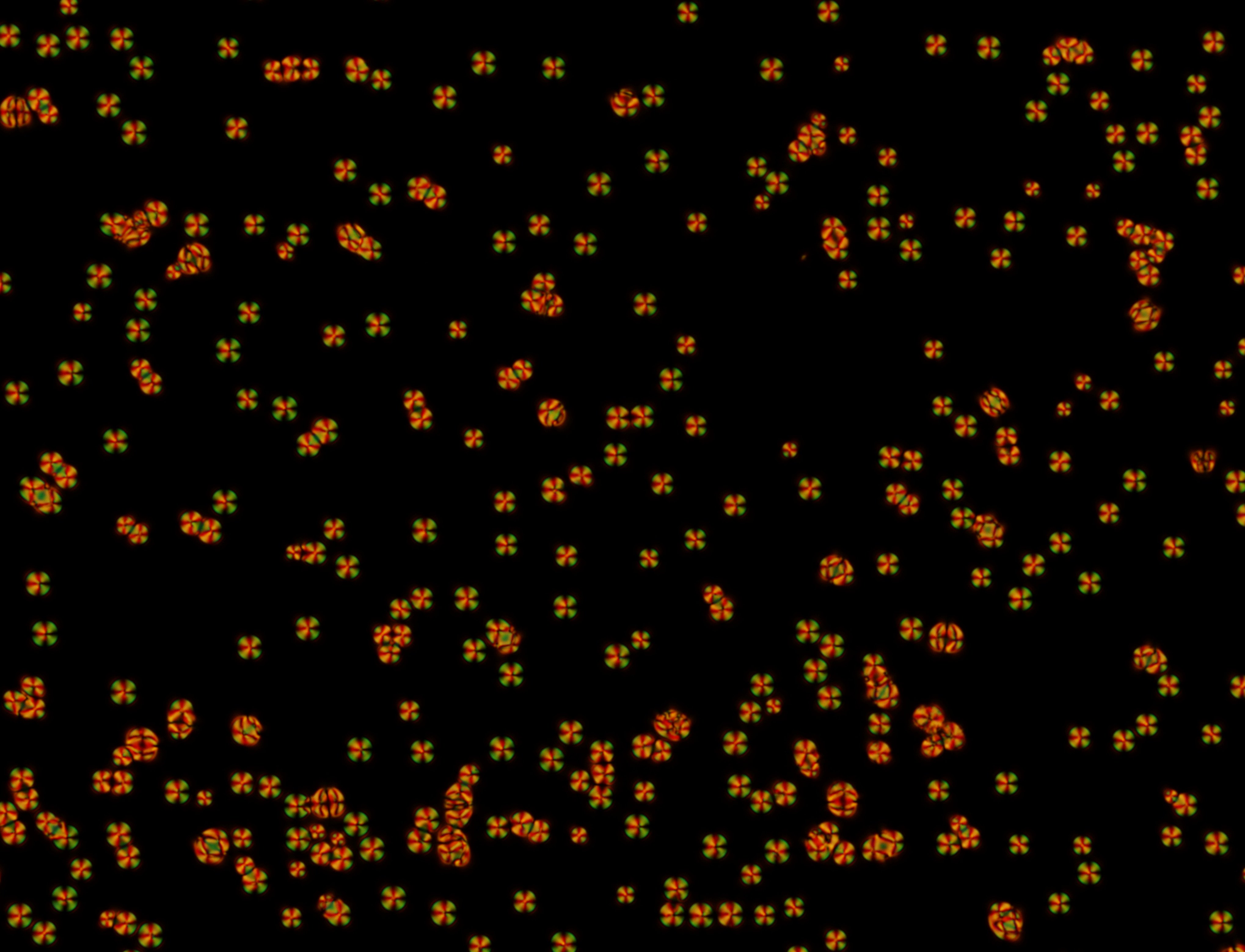

Supplement: Supplementary file 4 — Source Data [file 41467_2022_35443_MOESM4_ESM.zip › Source Data/Source Data Fig.2/Fig2b.jpg]

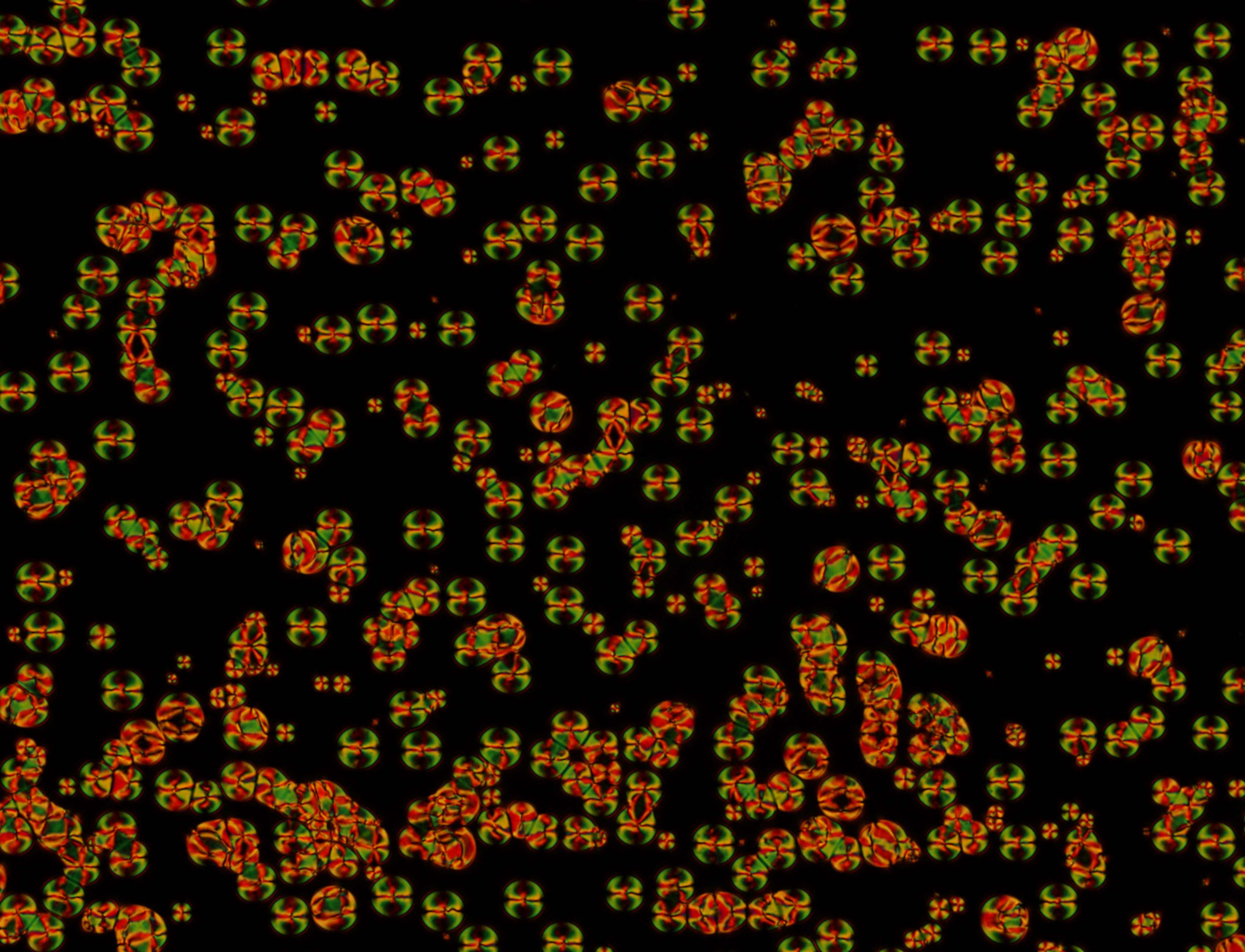

Supplement: Supplementary file 4 — Source Data [file 41467_2022_35443_MOESM4_ESM.zip › Source Data/Source Data Fig.2/Fig2c.jpg]

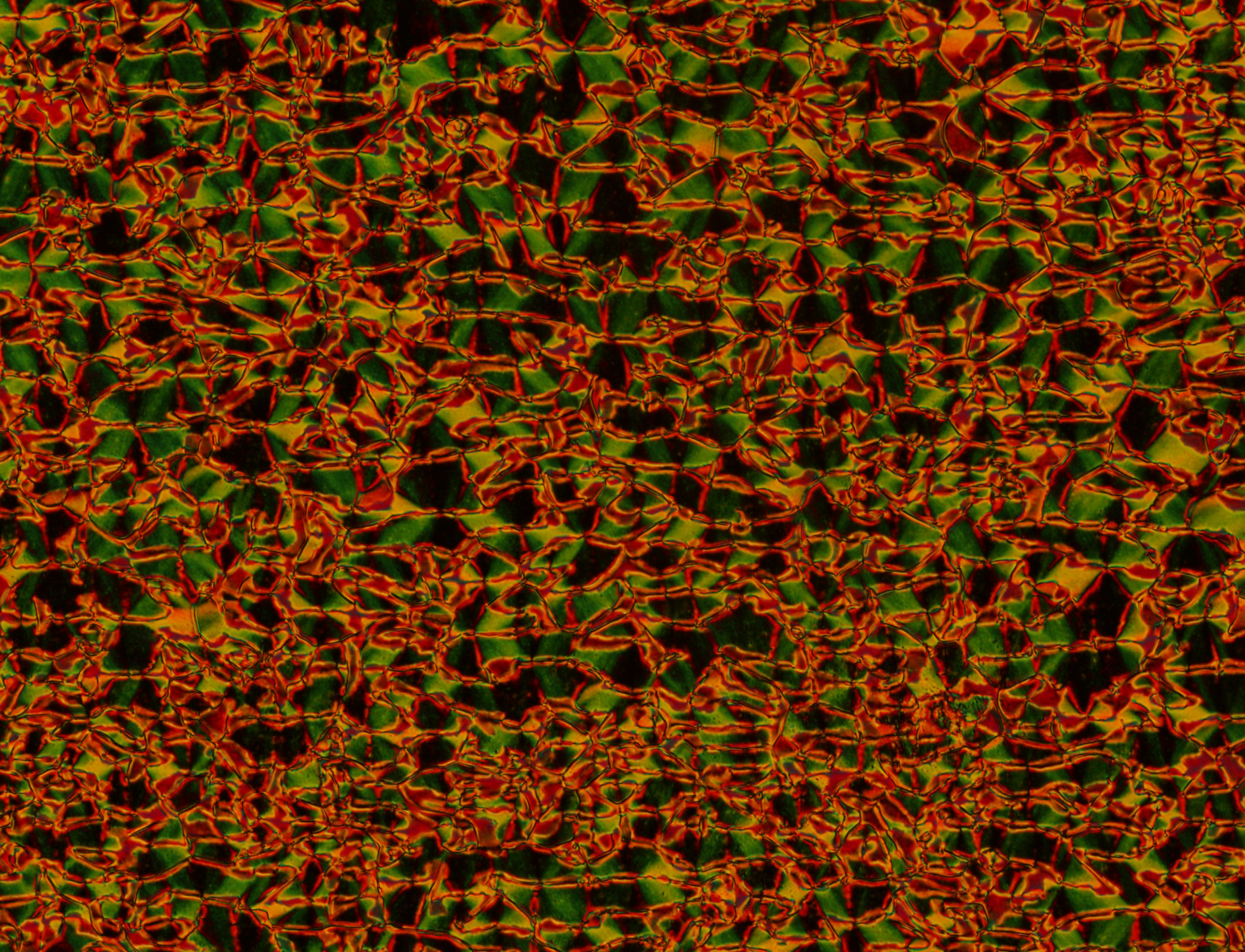

Supplement: Supplementary file 4 — Source Data [file 41467_2022_35443_MOESM4_ESM.zip › Source Data/Source Data Fig.2/Fig2d.jpg]

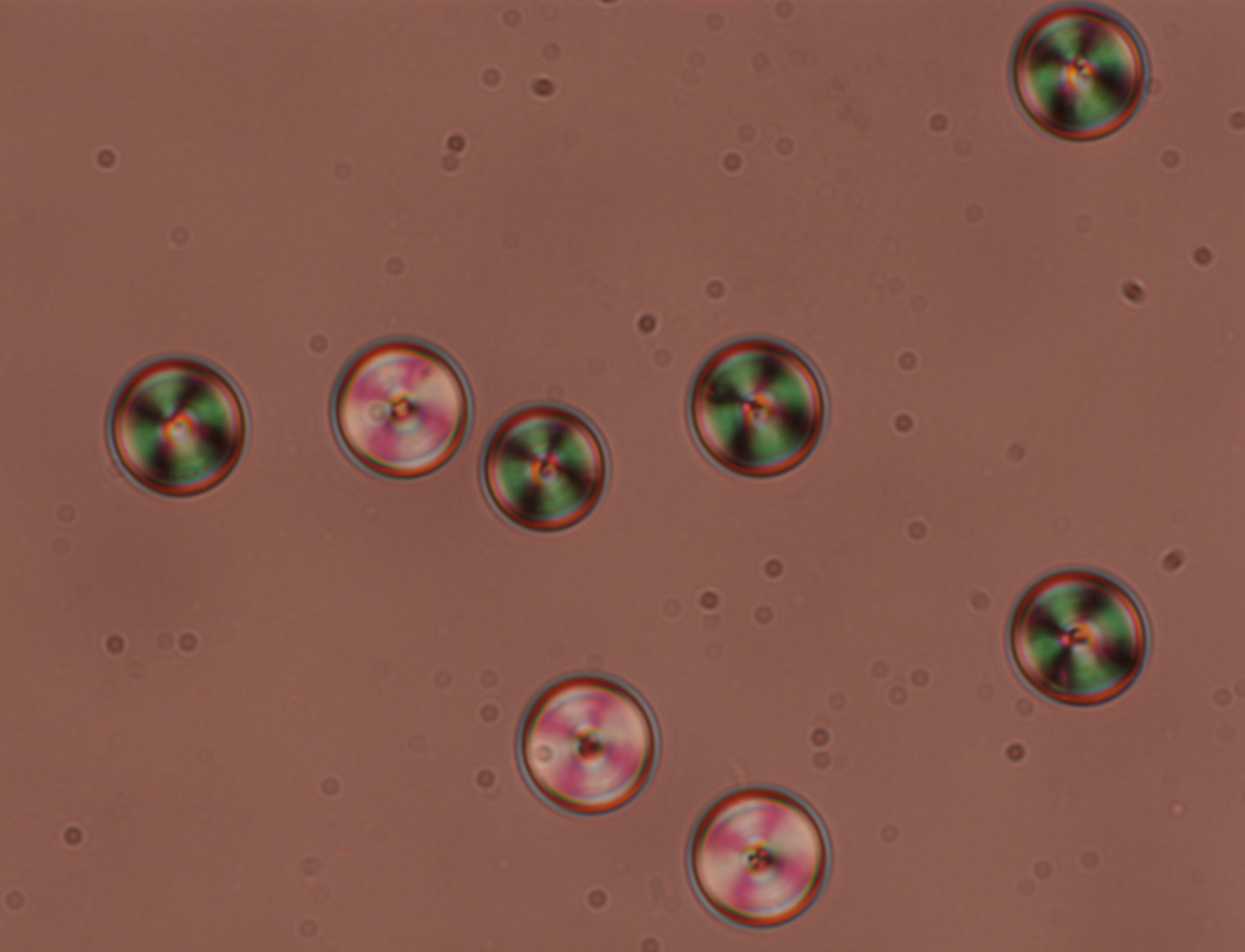

Supplement: Supplementary file 4 — Source Data [file 41467_2022_35443_MOESM4_ESM.zip › Source Data/Source Data Fig.3/Fig.3a.jpg]

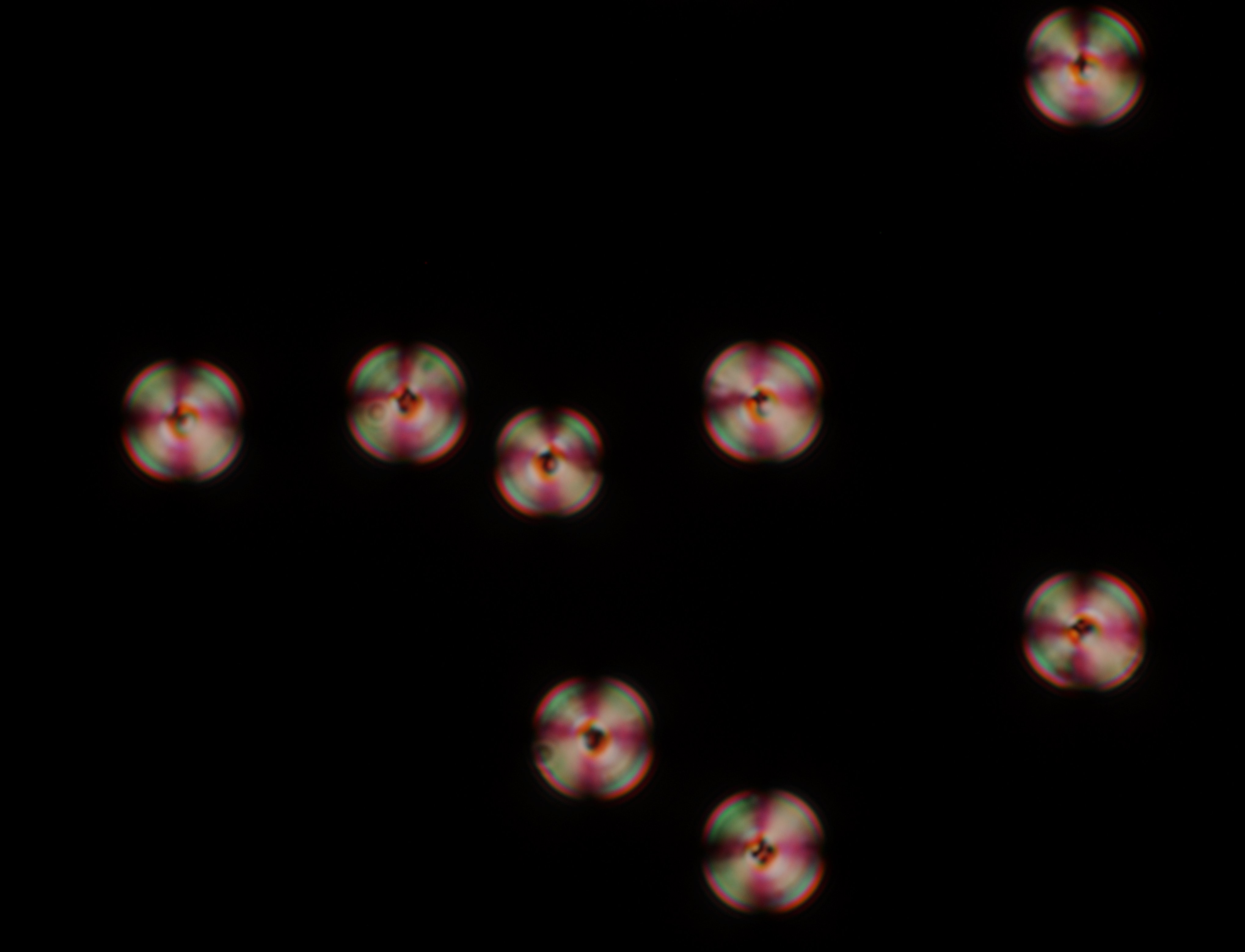

Supplement: Supplementary file 4 — Source Data [file 41467_2022_35443_MOESM4_ESM.zip › Source Data/Source Data Fig.3/Fig.3b.jpg]

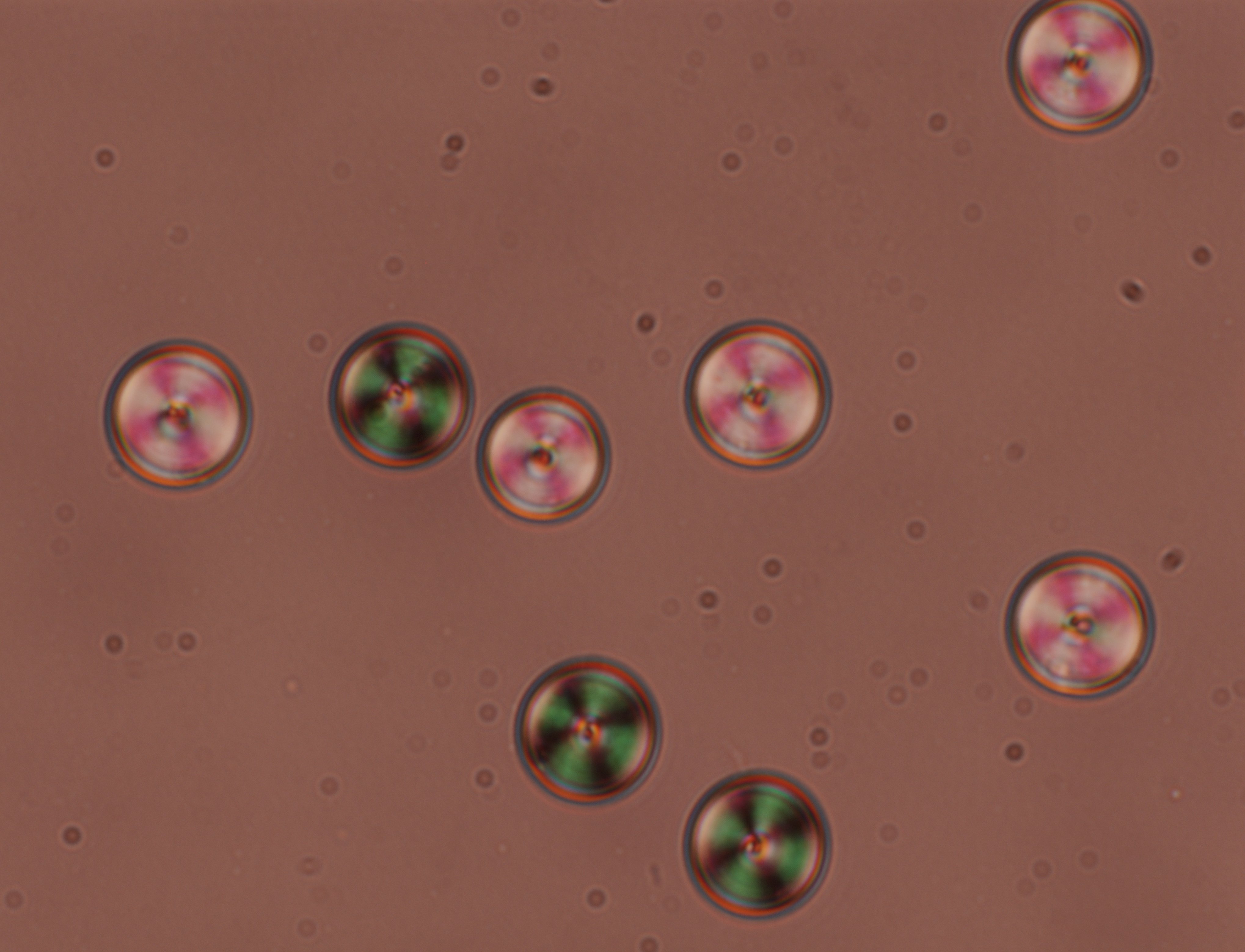

Supplement: Supplementary file 4 — Source Data [file 41467_2022_35443_MOESM4_ESM.zip › Source Data/Source Data Fig.3/Fig.3c .jpg]

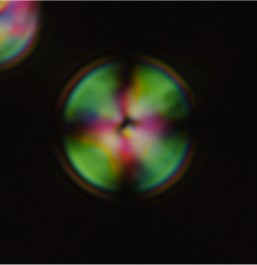

Supplement: Supplementary file 4 — Source Data [file 41467_2022_35443_MOESM4_ESM.zip › Source Data/Source Data Fig.3/Fig.3d.jpg]

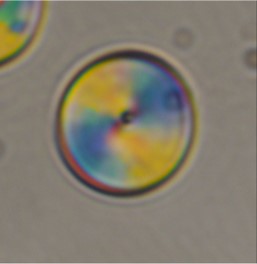

Supplement: Supplementary file 4 — Source Data [file 41467_2022_35443_MOESM4_ESM.zip › Source Data/Source Data Fig.3/Fig.3e.jpg]

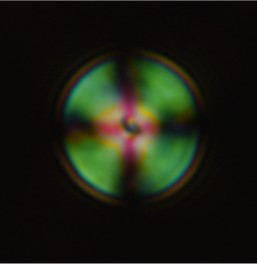

Supplement: Supplementary file 4 — Source Data [file 41467_2022_35443_MOESM4_ESM.zip › Source Data/Source Data Fig.3/Fig.3f.jpg]

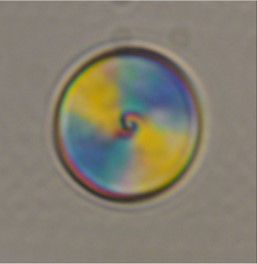

Supplement: Supplementary file 4 — Source Data [file 41467_2022_35443_MOESM4_ESM.zip › Source Data/Source Data Fig.3/Fig.3g.jpg]

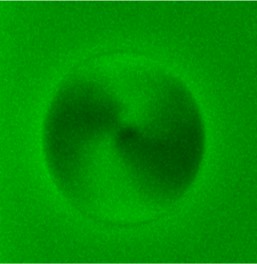

Supplement: Supplementary file 4 — Source Data [file 41467_2022_35443_MOESM4_ESM.zip › Source Data/Source Data Fig.3/Fig.3h.jpg]

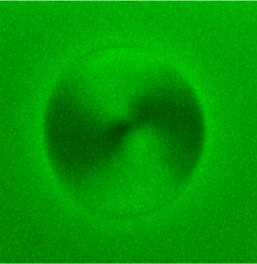

Supplement: Supplementary file 4 — Source Data [file 41467_2022_35443_MOESM4_ESM.zip › Source Data/Source Data Fig.3/Fig.3j.jpg]

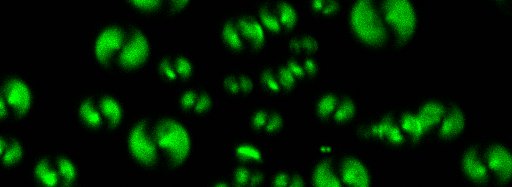

Supplement: Supplementary file 4 — Source Data [file 41467_2022_35443_MOESM4_ESM.zip › Source Data/Source Data Fig.4/Fig4a.jpg]

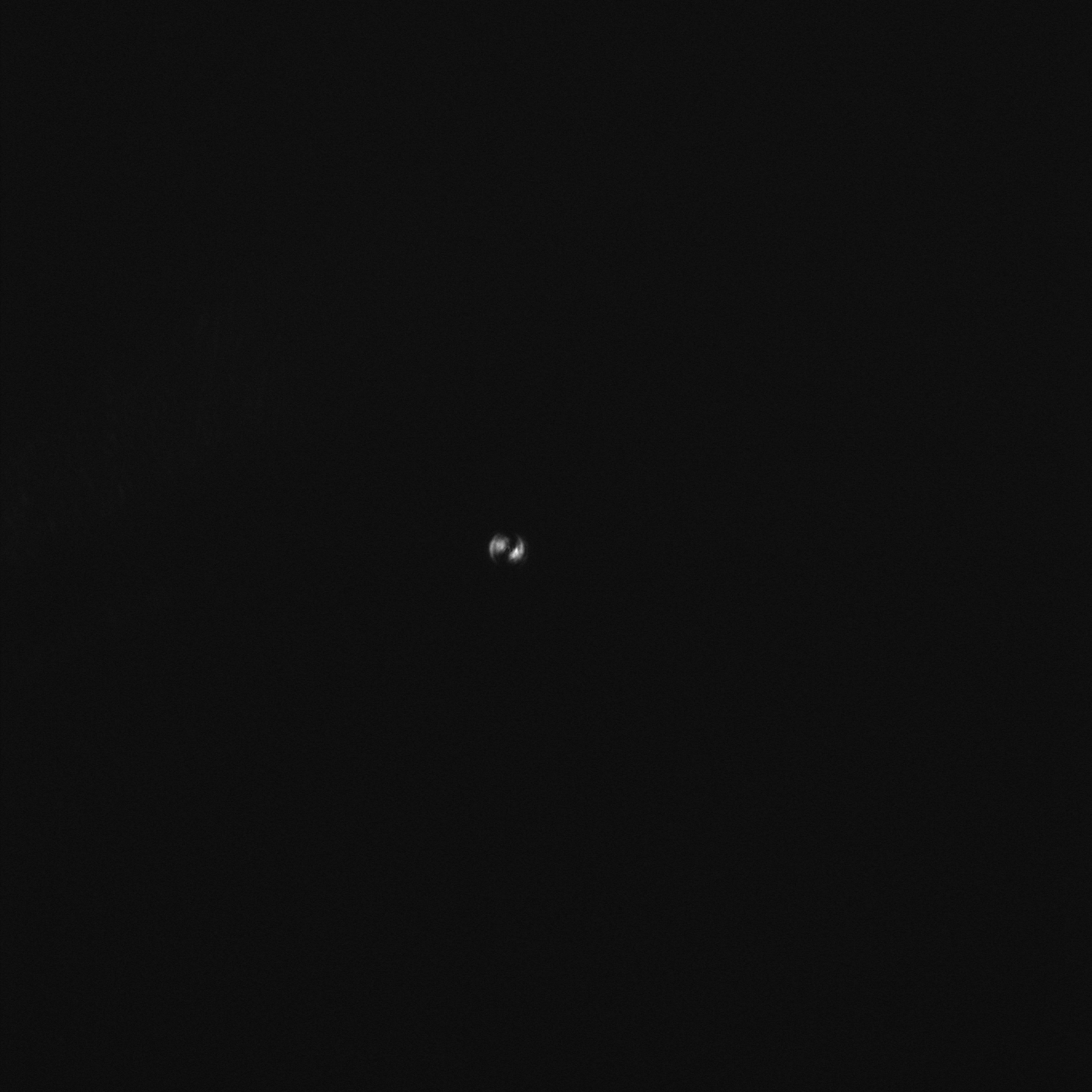

Supplement: Supplementary file 4 — Source Data [file 41467_2022_35443_MOESM4_ESM.zip › Source Data/Source Data Fig.4/Fig4d.bmp]

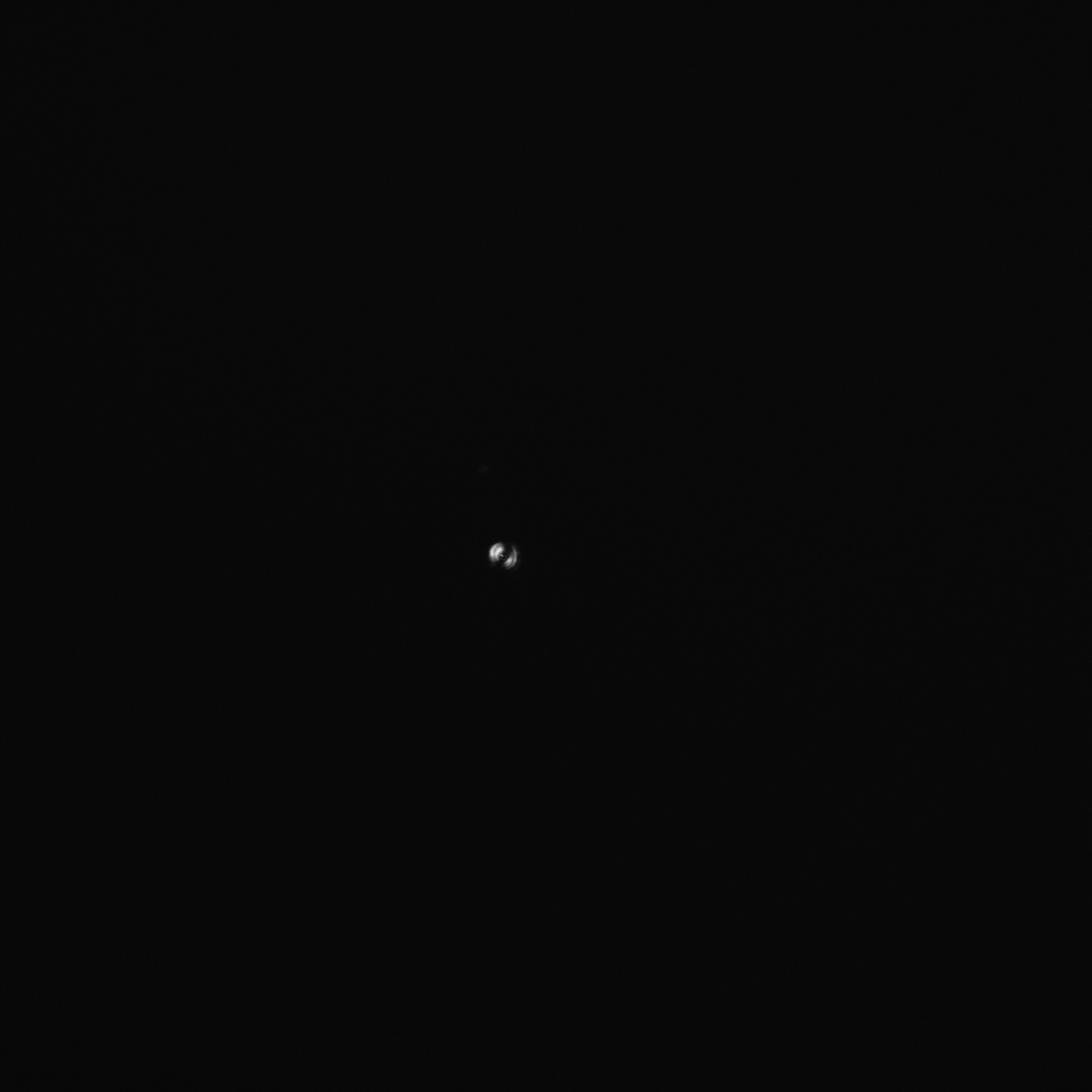

Supplement: Supplementary file 4 — Source Data [file 41467_2022_35443_MOESM4_ESM.zip › Source Data/Source Data Fig.4/Fig4e.bmp]

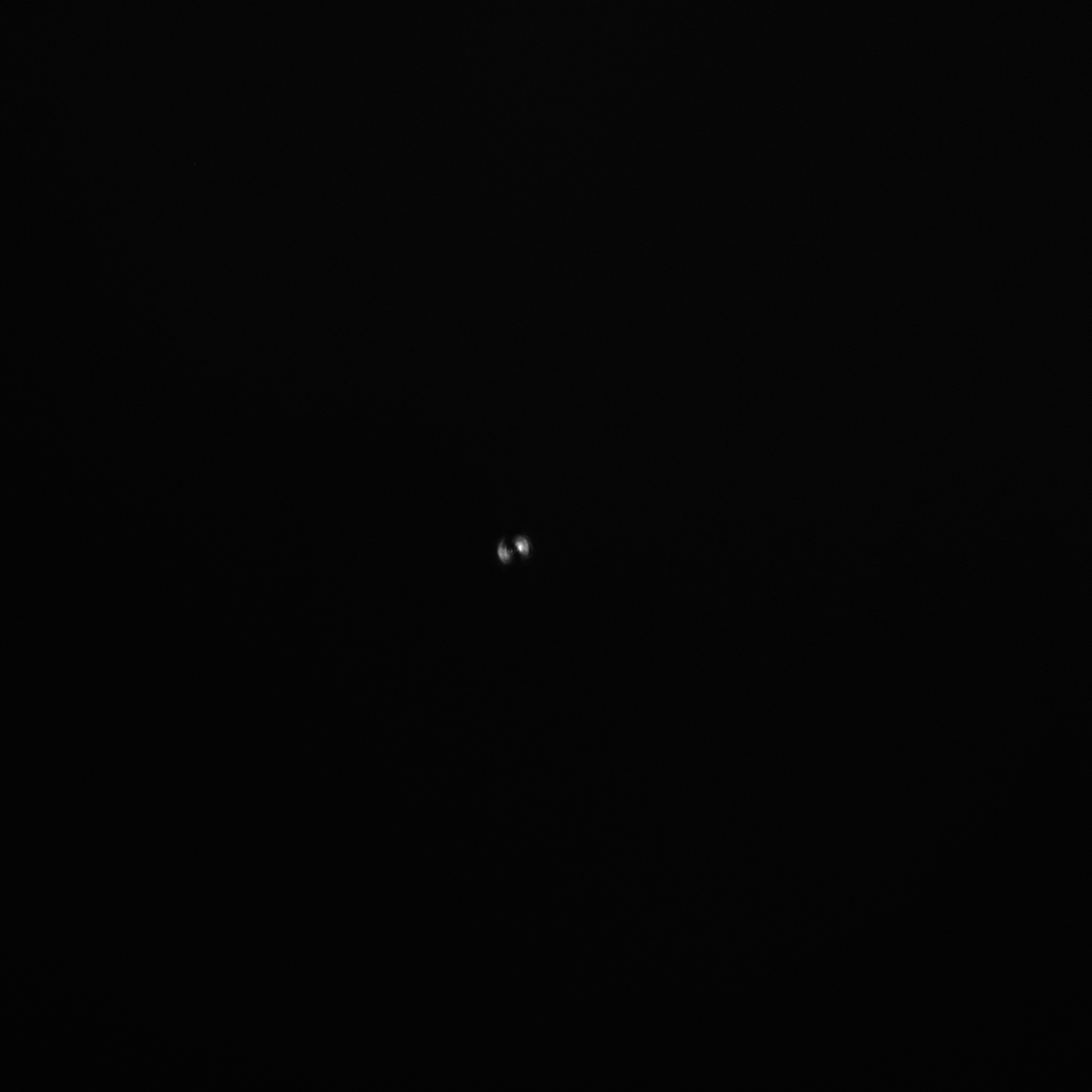

Supplement: Supplementary file 4 — Source Data [file 41467_2022_35443_MOESM4_ESM.zip › Source Data/Source Data Fig.4/Fig4f.bmp]

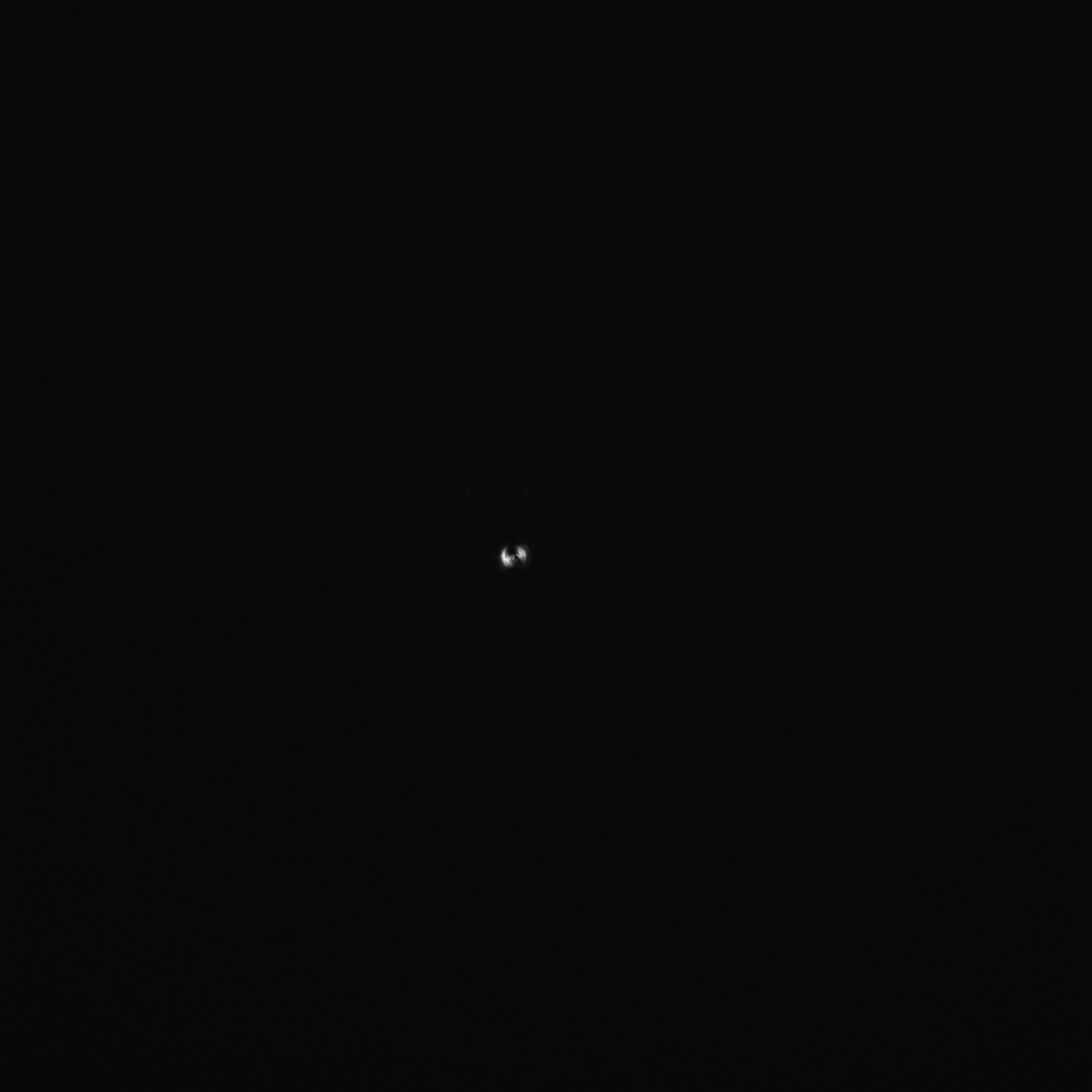

Supplement: Supplementary file 4 — Source Data [file 41467_2022_35443_MOESM4_ESM.zip › Source Data/Source Data Fig.4/Fig4g.bmp]

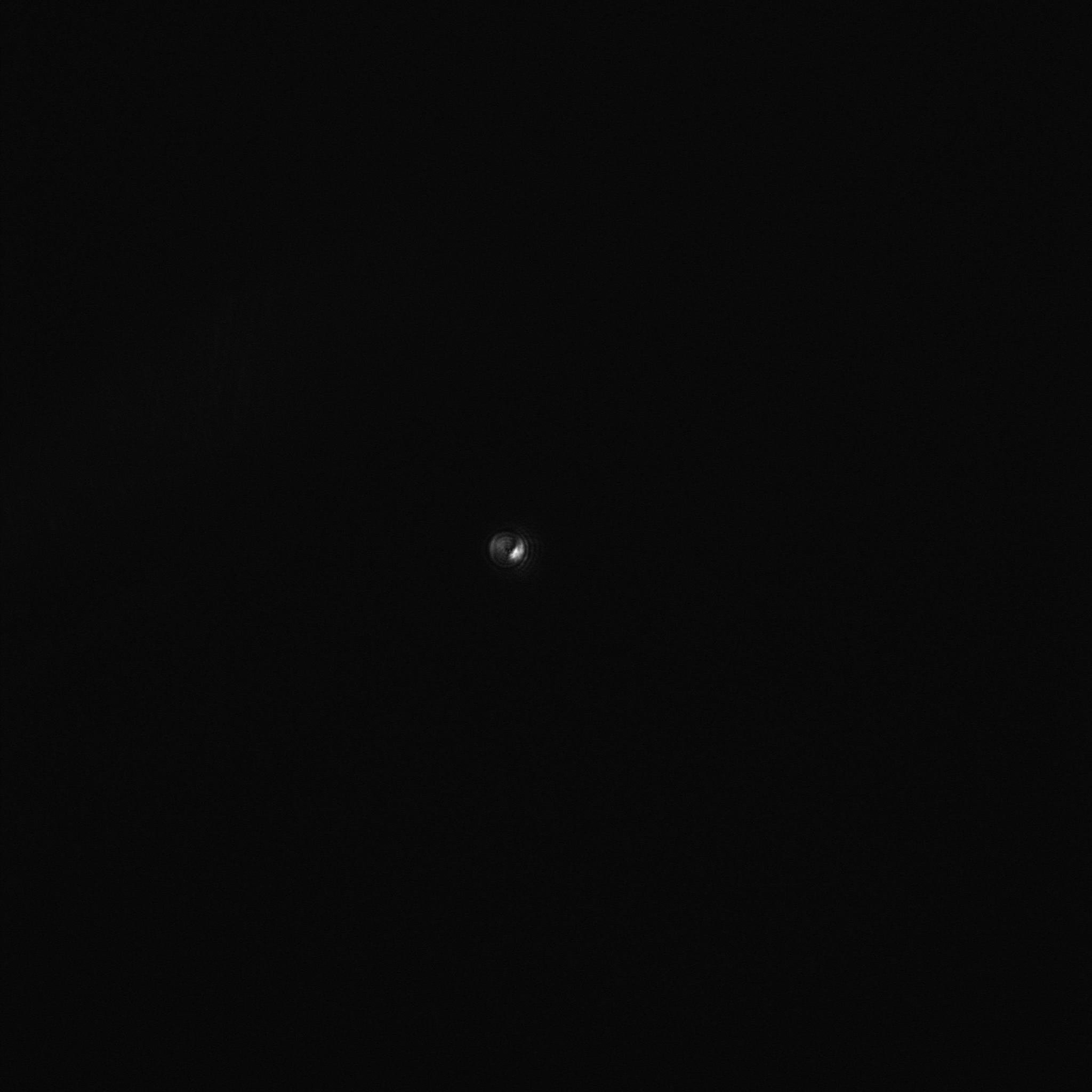

Supplement: Supplementary file 4 — Source Data [file 41467_2022_35443_MOESM4_ESM.zip › Source Data/Source Data Fig.4/Fig4h.bmp]

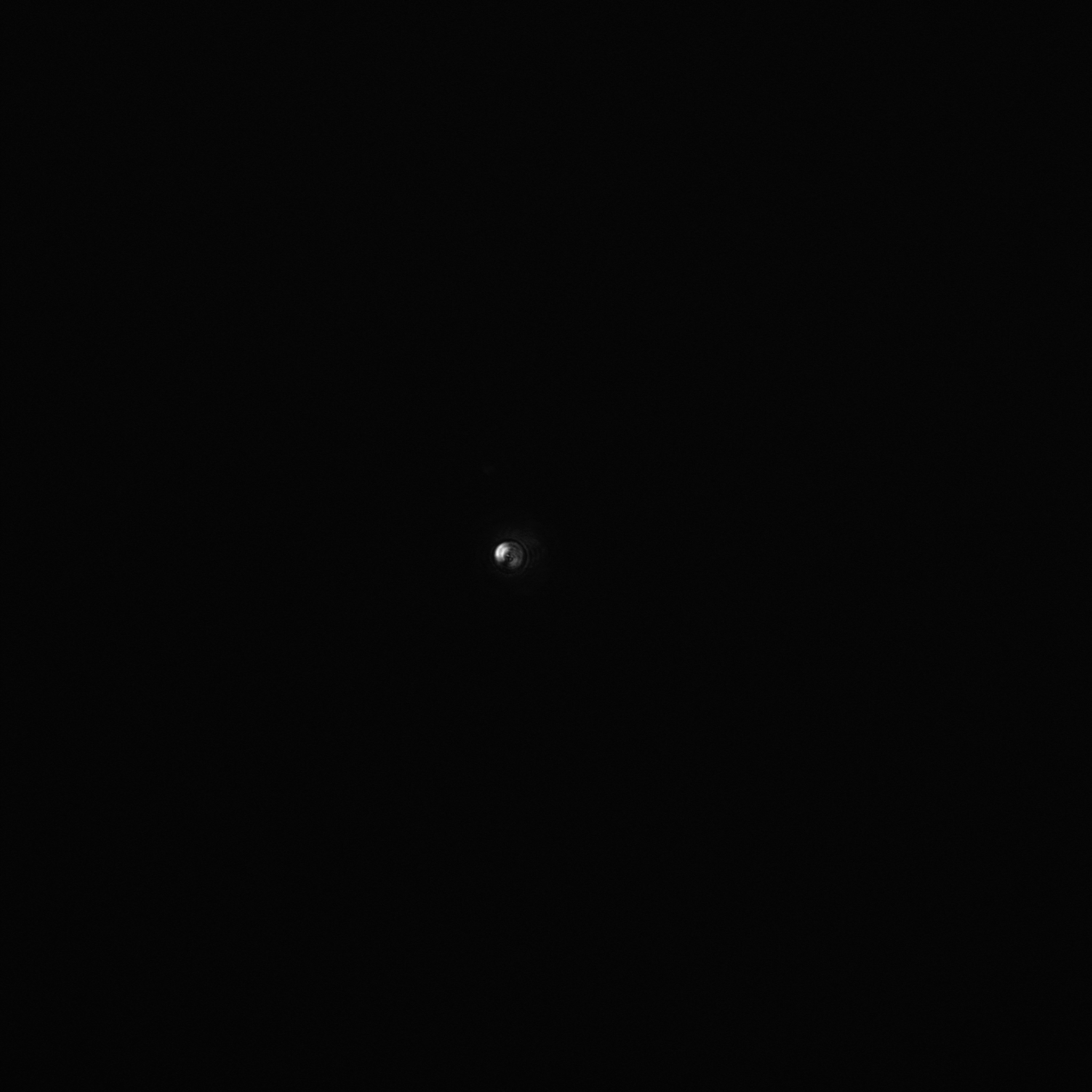

Supplement: Supplementary file 4 — Source Data [file 41467_2022_35443_MOESM4_ESM.zip › Source Data/Source Data Fig.4/Fig4i.bmp]

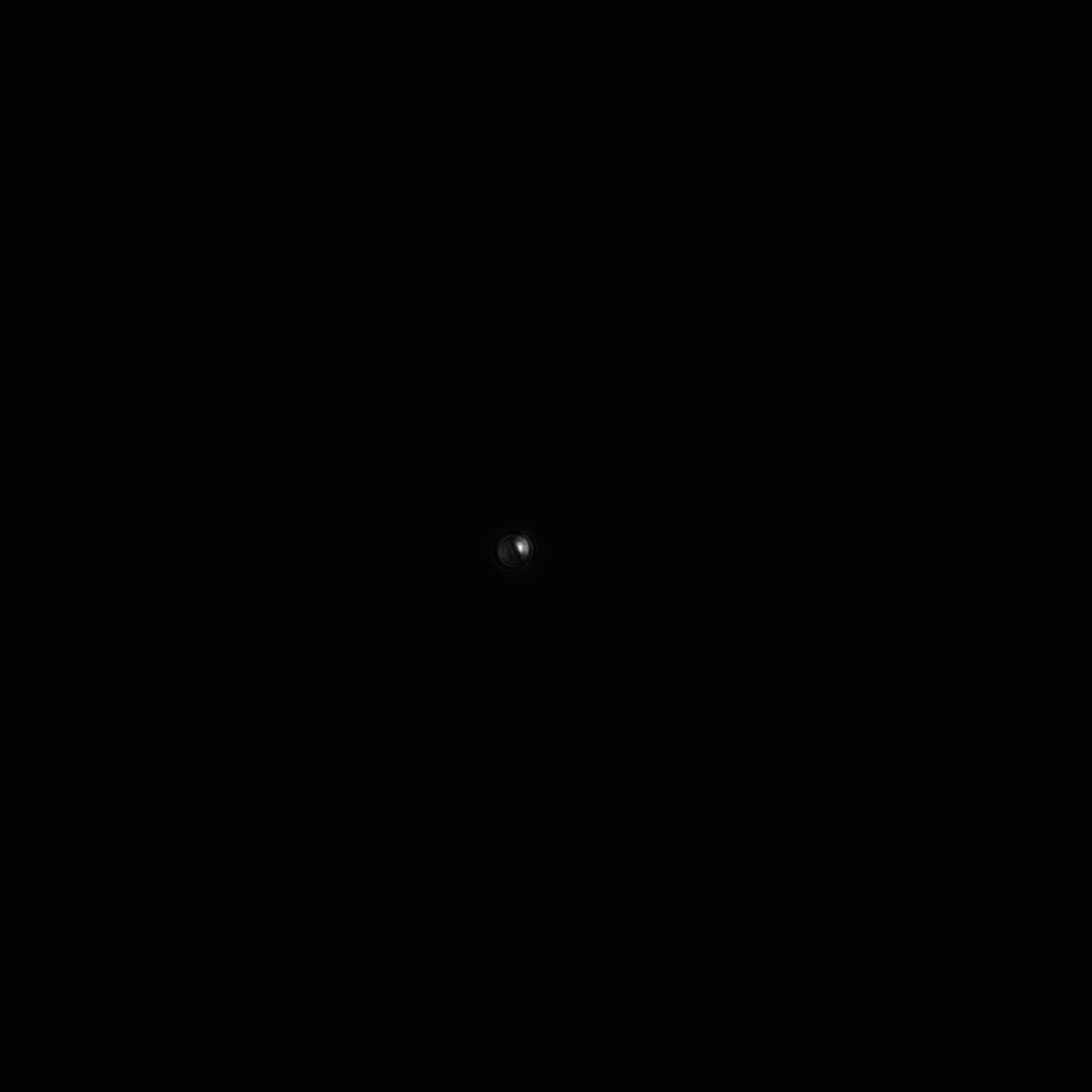

Supplement: Supplementary file 4 — Source Data [file 41467_2022_35443_MOESM4_ESM.zip › Source Data/Source Data Fig.4/Fig4j.bmp]

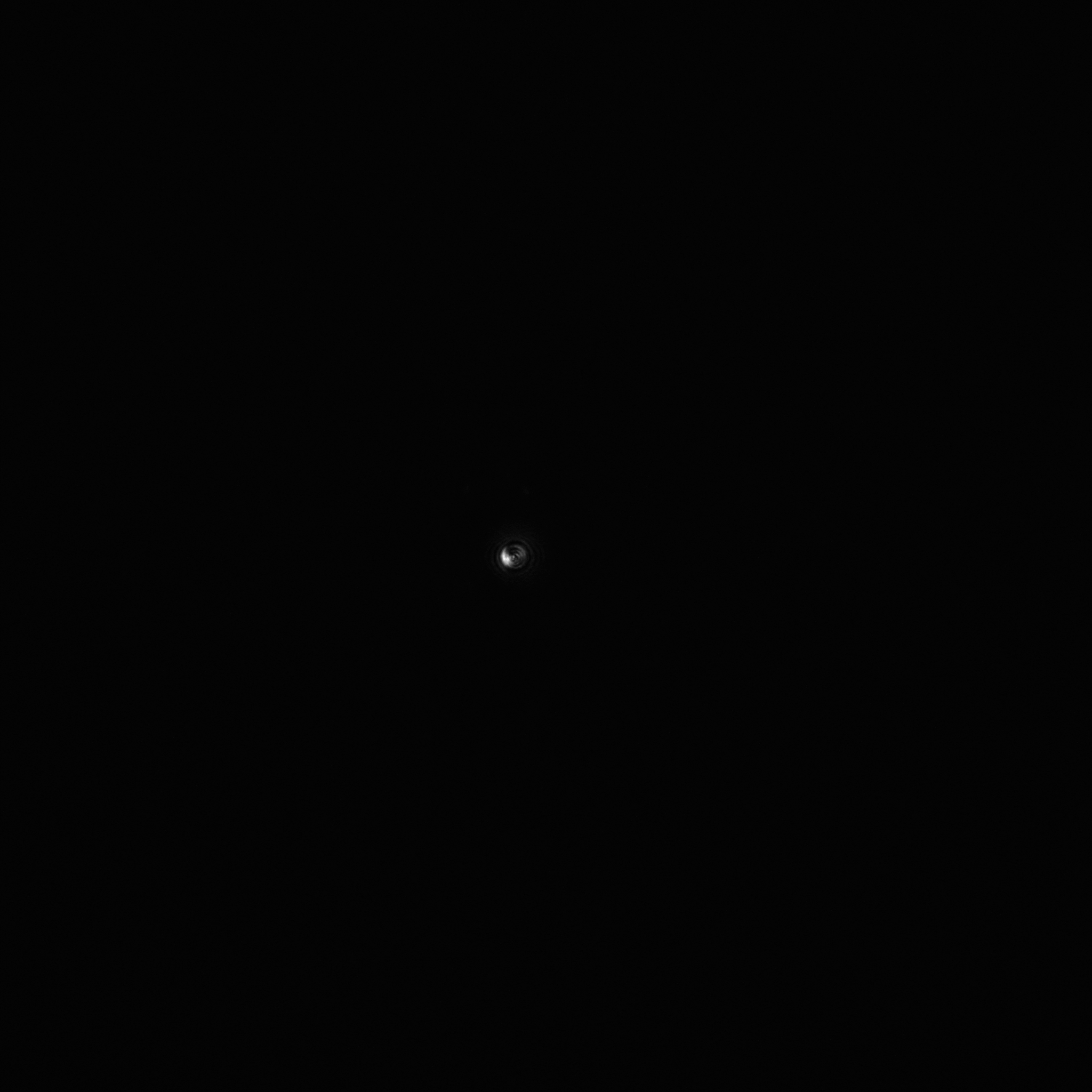

Supplement: Supplementary file 4 — Source Data [file 41467_2022_35443_MOESM4_ESM.zip › Source Data/Source Data Fig.4/Fig4k.bmp]

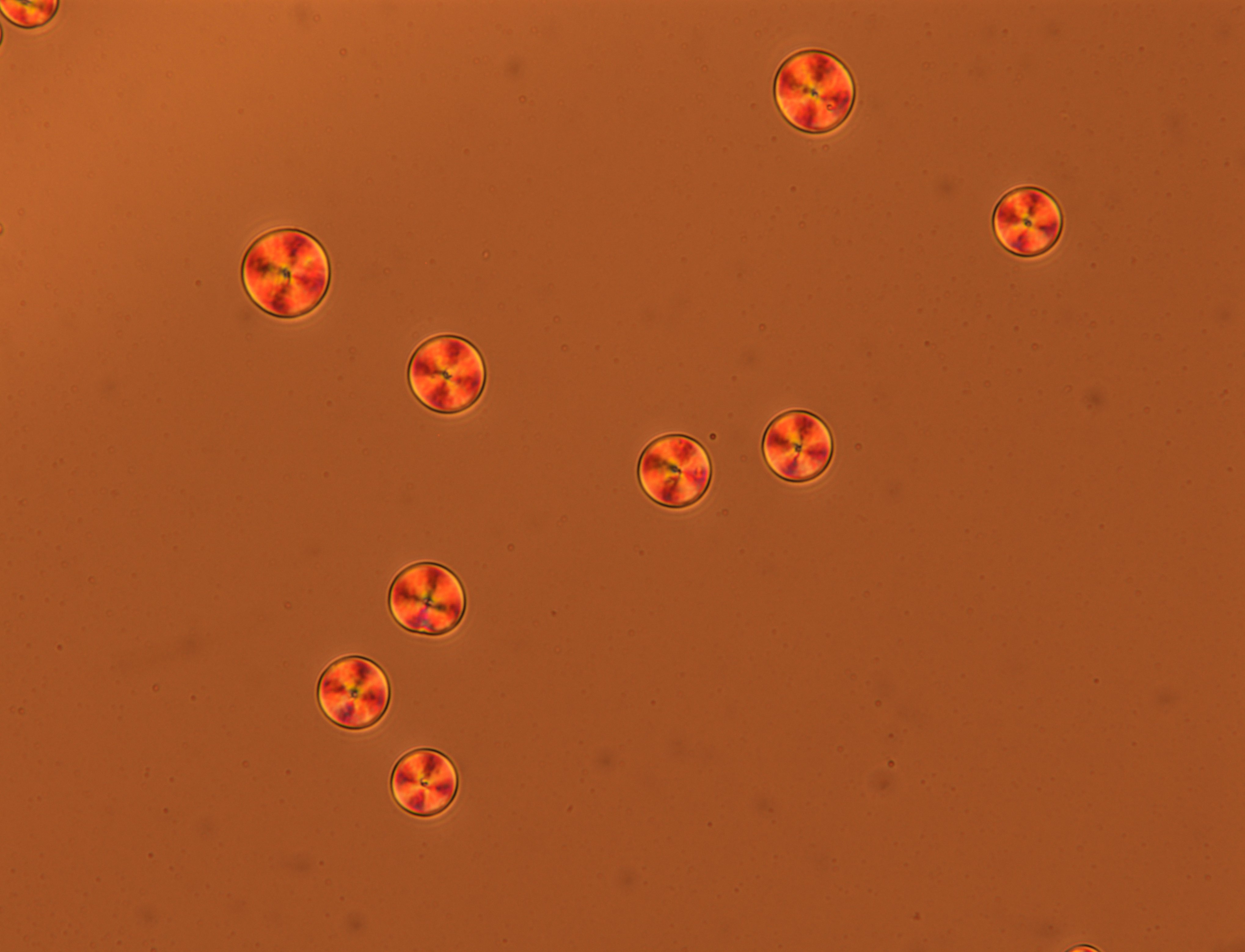

Supplement: Supplementary file 4 — Source Data [file 41467_2022_35443_MOESM4_ESM.zip › Source Data/Source Data Fig.5/Fig.5a.jpg]

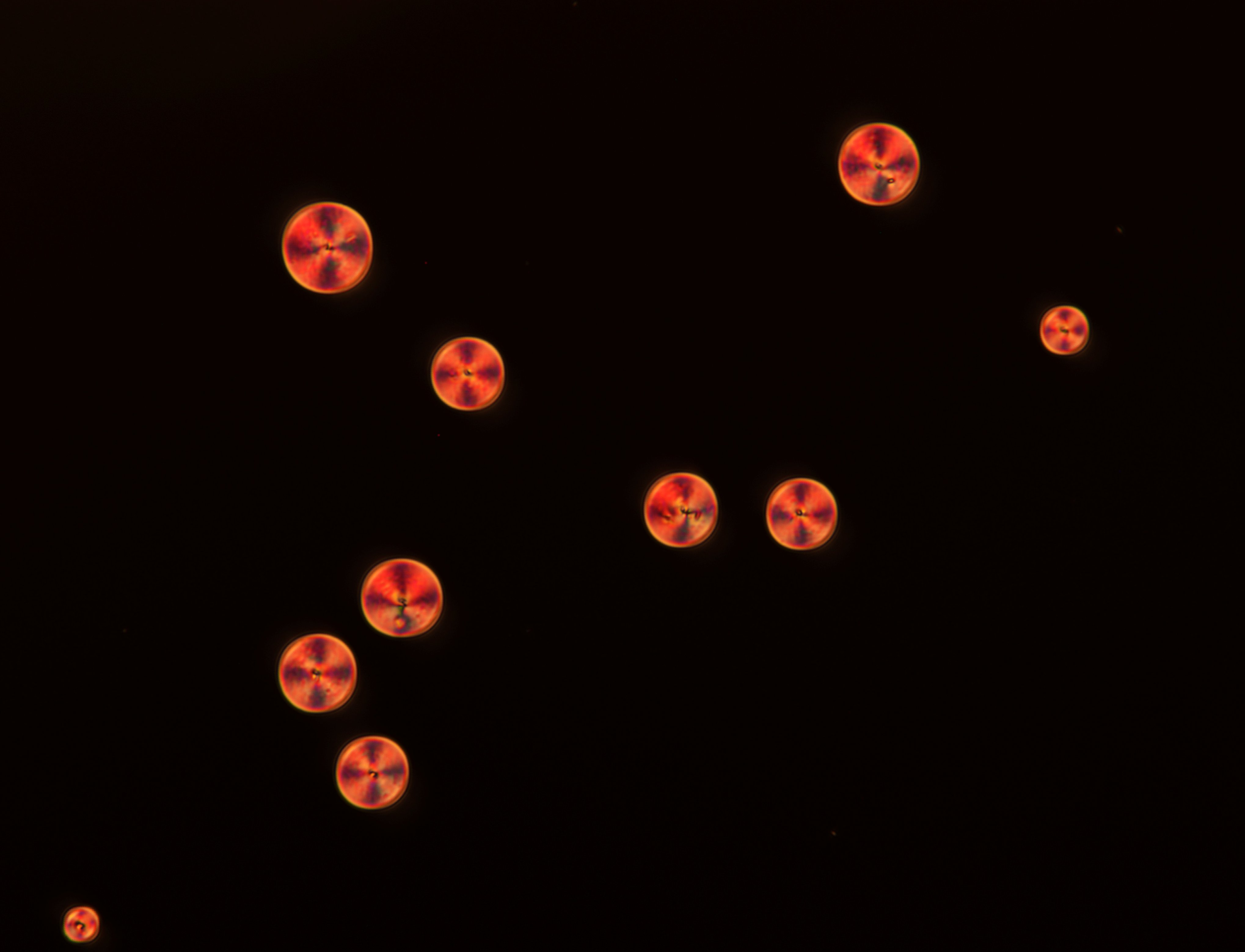

Supplement: Supplementary file 4 — Source Data [file 41467_2022_35443_MOESM4_ESM.zip › Source Data/Source Data Fig.5/Fig.5b.jpg]

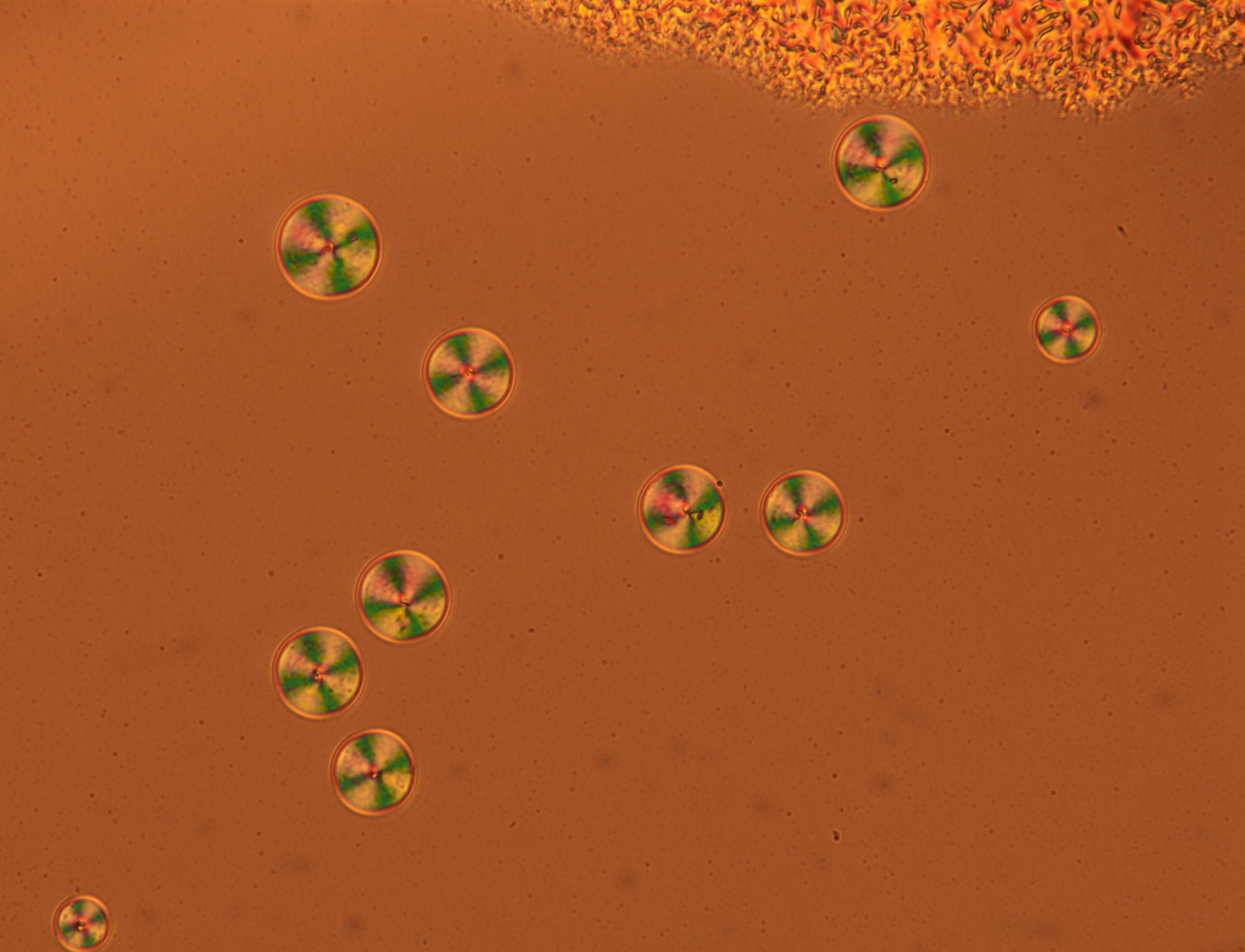

Supplement: Supplementary file 4 — Source Data [file 41467_2022_35443_MOESM4_ESM.zip › Source Data/Source Data Fig.5/Fig.5c.jpg]

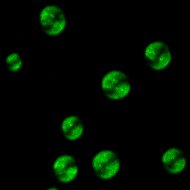

Supplement: Supplementary file 4 — Source Data [file 41467_2022_35443_MOESM4_ESM.zip › Source Data/Source Data Fig.5/Fig.5d.jpg]

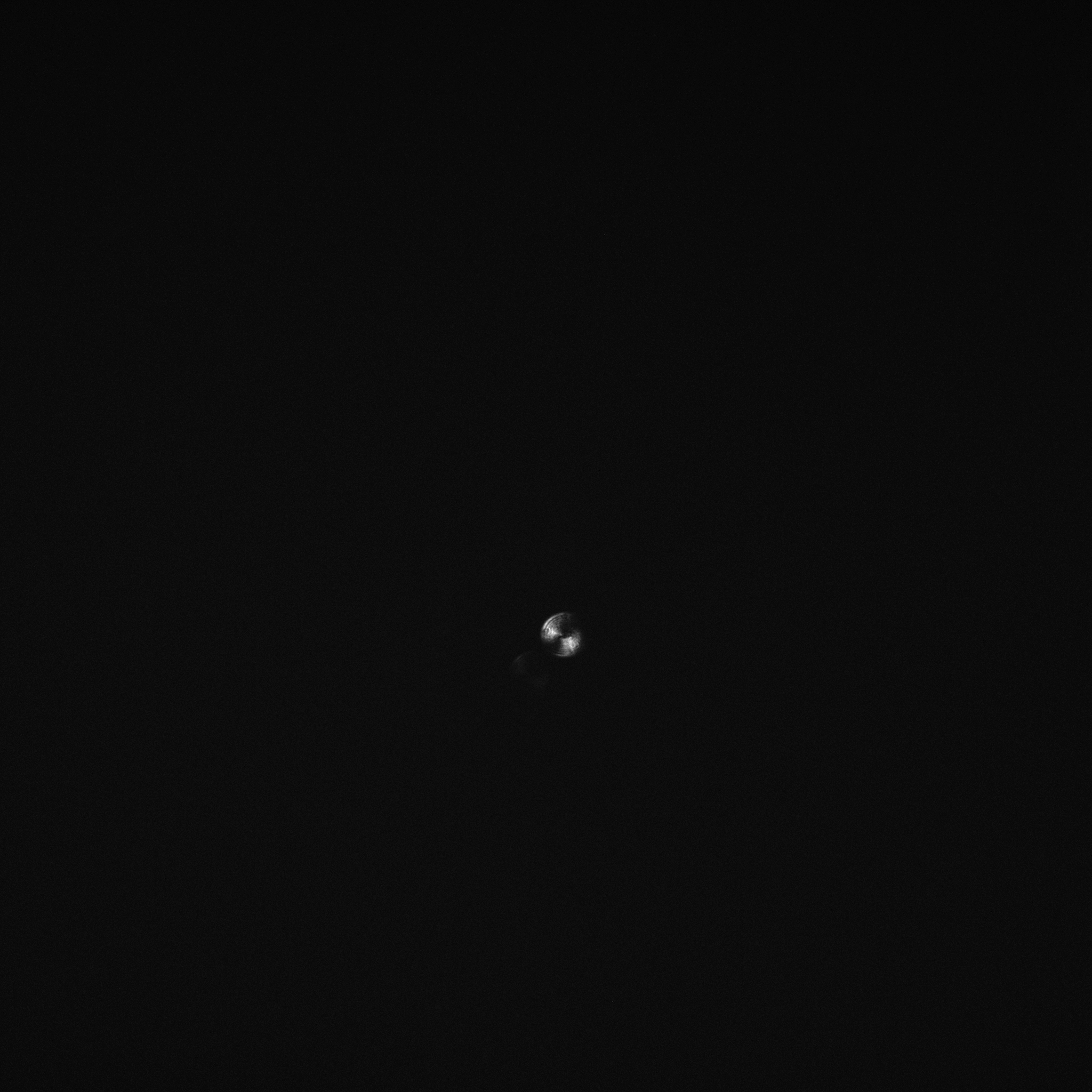

Supplement: Supplementary file 4 — Source Data [file 41467_2022_35443_MOESM4_ESM.zip › Source Data/Source Data Fig.5/Fig.5e.bmp]

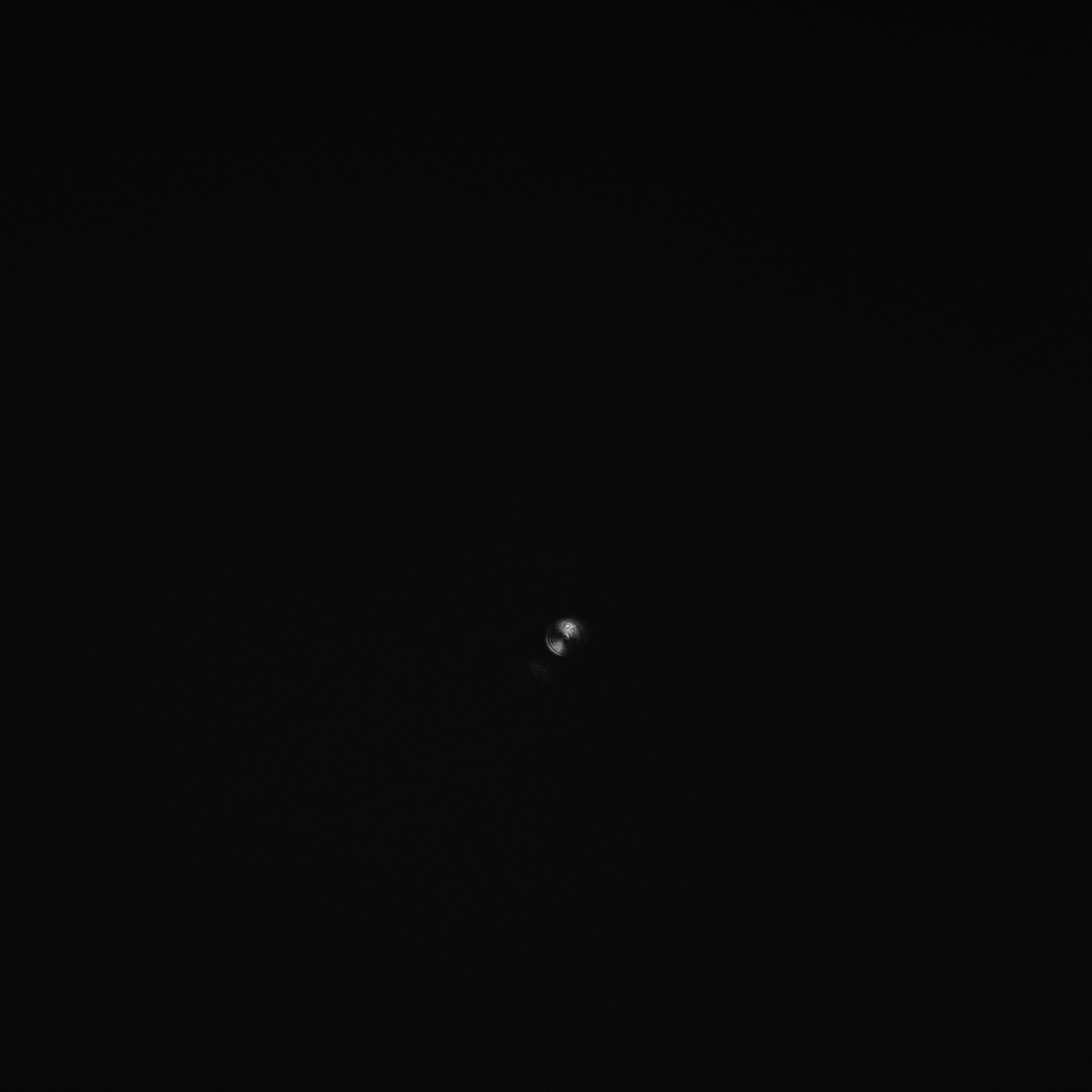

Supplement: Supplementary file 4 — Source Data [file 41467_2022_35443_MOESM4_ESM.zip › Source Data/Source Data Fig.5/Fig.5f.bmp]

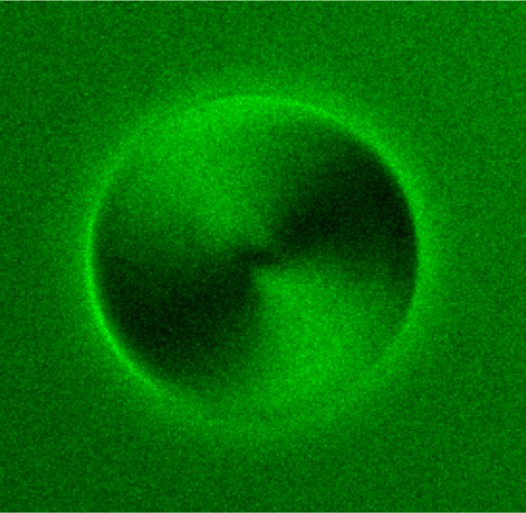

Supplement: Supplementary file 4 — Source Data [file 41467_2022_35443_MOESM4_ESM.zip › Source Data/Source Data Fig.5/Fig.5g.jpg]
